# Assessment of the Diagnostic Efficiency of a Liquid Biopsy Assay for Early Detection of Gastric Cancer

**DOI:** 10.1001/jamanetworkopen.2021.21129

**Published:** 2021-08-24

**Authors:** Daisuke Izumi, Zhongxu Zhu, Yuetong Chen, Shusuke Toden, Xinying Huo, Mitsuro Kanda, Takatsugu Ishimoto, Dongying Gu, Miaomiao Tan, Yasuhiro Kodera, Hideo Baba, Wei Li, Jinfei Chen, Xin Wang, Ajay Goel

**Affiliations:** 1 Center for Gastrointestinal Research, Baylor Scott & White Research Institute, Baylor University Medical Center, Dallas, Texas; 2 Department of Gastroenterological Surgery, Graduate School of Medical Sciences, Kumamoto University, Kumamoto, Japan; 3 Department of Surgery, Japanese Community Health Care Organization Kumamoto General Hospital, Kumamoto, Japan; 4 Department of Biomedical Sciences, City University of Hong Kong, Hong Kong SAR, China; 5 Department of Oncology, Nanjing First Hospital, Nanjing Medical University, Nanjing, China; 6 Department of Gastroenterological Surgery, Nagoya University Graduate School of Medicine, Nagoya, Japan; 7 Shenzhen Research Institute, City University of Hong Kong, Shenzhen, China; 8 The International Research Center for Medicine Sciences, Kumamoto University, Kumamoto, Japan; 9 Department of Biological Chemistry, School of Medicine, University of California, Irvine; 10 Cancer Center, Taikang Xianlin Drum Tower Hospital, Nanjing University School of Medicine, Nanjing, China; 11 Collaborative Innovation Center for Cancer Personalized Medicine, Nanjing Medical University, Nanjing, China; 12 Department of Molecular Diagnostics and Experimental Therapeutics, Beckman Research Institute of City of Hope, Monrovia; 13 City of Hope Comprehensive Cancer Center, Duarte, California; 14 Charles A. Sammons Cancer Center, Baylor University Medical Center, Dallas, Texas

## Abstract

**Importance:**

Noninvasive detection of early-stage disease is a key strategy for reducing gastric cancer (GC)-associated patient mortality.

**Objective:**

To establish a novel, noninvasive, microRNA (miRNA)-based signature for the early detection of GC using a comprehensive biomarker discovery approach with retrospective and prospective validation.

**Design, Setting, and Participants:**

This diagnostic study was conducted in 4 phases using publicly available genome sequences and tissue samples from patients at an academic medical center in Japan, and validated with retrospective multicenter cohorts of patients with GC. Three tissue miRNA data sets were used to identify a miRNA signature that discriminated GC vs normal tissues. The robustness of this signature was assessed in serum from 2 retrospective cohorts of patients with GC. A risk-scoring model was derived, then the performance of the miRNA signature was evaluated in a prospective cohort of patients with GC. The robustness of the miRNA signature was compared with current blood-based markers, and a cost-effectiveness analysis of the miRNA signature against the current practice of endoscopy was performed. All clinical samples used for this study were collected and data analyzed between April 1997 and March 2018.

**Main Outcomes and Measures:**

Assessment of diagnostic efficiency on the basis of area under the curve (AUC), specificity, and sensitivity.

**Results:**

The data sets for the genome-wide expression profiling analysis stage included 598 total patient samples (284 [55.4%] from men; mean [SE] patient age, 65.7 [0.5] years). The resulting 10-miRNA signature was validated in 2 retrospective GC serum cohorts (586 patients; 348 [59.4%] men, mean [SE] age, 66.0 [0.7] years), which led to the establishment of a 5-miRNA signature (AUC, 0.90; 95% CI, 0.85-0.94) that also exhibited high levels of diagnostic performance in patients with stage I disease (AUC, 0.89; 95% CI, 0.83-0.94). A risk-scoring model was derived and the assay was optimized to a minimal number of miRNAs. The performance of the resulting 3-miRNA signature was then validated in a prospective cohort of patients with GC (349 patients; 124 [70.5%] men, median [range] age, 66.0 [0.66] years). The final 3-miRNA signature (miR-18a, miR-181b, and miR-335) exhibited high diagnostic accuracy in all stages of patients (AUC, 0.86; 95% CI 0.83-0.90), including in patients with stage I disease (AUC, 0.85; 95% CI, 0.79-0.91). Furthermore, this miRNA signature was superior to currently used blood markers and outperformed the endoscopic screening in a cost-effectiveness analysis (incremental cost-effectiveness ratio, CNY ¥16162.5 per quality-adjusted life-year [USD $2304.80 per quality-adjusted life-year]).

**Conclusions and Relevance:**

These results suggest the potential clinical significance of the 3-miRNA signature as a noninvasive, cost-effective, and facile assay for the early detection of GC.

## Key Points

**Question:**

Can circulating microRNAs be used to derive clinically significant noninvasive diagnostic biomarkers for gastric cancer?

**Findings:**

This diagnostic study used a multistep and comprehensive biomarker discovery approach to establish a novel, noninvasive, microRNA-based signature for the early detection of gastric cancer, which was retrospectively and prospectively validated in multicenter patient cohorts.

**Meaning:**

For patients with gastric cancer and individuals with a high risk for gastric cancer, a microRNA-based signature may improve the early detection of gastric cancer.

## Teaser

This diagnostic study describes the development and validation of a microRNA-based liquid biopsy assay for early detection of gastric cancer.

## Introduction

Gastric cancer (GC) is the fourth most-commonly diagnosed cancer, and the third leading cause of cancer-associated mortality worldwide.^1^ Despite improvements in treatment modalities, the prognosis for advanced GC following curative resection remains poor.^2^ By contrast, patients diagnosed with early-stage GC have a favorable prognosis, underscoring the paradigm that identification at earlier stages remains an attractive strategy for reducing GC-associated patient mortality. Although endoscopic surveillance accompanied by pathological examination of tissue biopsies remains the criterion standard for screening and diagnosis of patients with asymptomatic GC, this approach is inadequate for screening the average-risk population because of its invasive nature and high expense. Thus, the use of liquid biopsy-based, noninvasive cancer biomarkers has become increasingly desirable, and several promising molecular biomarkers have been identified in blood, urine, and gastric juice.^3,4,5^ Despite these advances, serum levels of carcinoembryonic antigen (CEA) and cancer antigen 19-9 (CA19-9) are the only markers currently available for clinical use. However, these biomarkers are typically not elevated in early-stage GCs and are therefore inadequate at identifying patients with early-stage GC.

MicroRNAs (miRNAs) are small noncoding RNAs that regulate the expression of target genes through transcriptional interference or inhibition and are involved in most biological events, including tumorigenesis.^6^ Over the last decade, tumor-derived miRNAs in peripheral blood have emerged as promising disease biomarkers^7^; however, considering tumor heterogeneity, the diagnostic potential of individual miRNAs appears to be limited.^8,9^ To overcome this challenge, recent studies suggested that a signature-based approach that combines the expression changes of multiple circulating miRNAs could be superior and more robust than using individual circulating miRNAs.^10,11^ However, a limited number of biomarker signatures are available and their diagnostic accuracy remains inadequate for diagnosing patients with early-stage GC.^12,13,14,15^ Herein, we established a novel, noninvasive, miRNA-based signature for the early detection of GC using a systematic and comprehensive biomarker discovery approach, which was then evaluated for its robustness retrospective and prospective in multicenter patient validation cohorts.

## Methods

### Study Design and Participants

We analyzed more than 1900 tissue and serum specimens from patients with GC, adjacent normal tissues, and healthy participants across 4 phases for this study. Study phases included a biomarker discovery phase, a tissue validation phase, a retrospective serum validation phase, and a prospective serum performance evaluation phase (eFigure 1 in the Supplement). Written informed consent was obtained from all participants, and the institutional review boards of all participating institutions approved the study. This study followed the Standards for Reporting of Diagnostic Accuracy (STARD) reporting guideline.

The biomarker discovery cohort (436 GC tissues and 41 adjacent normal mucosae from The Cancer Genome Atlas [TCGA]) was analyzed to identify miRNA candidates. Subsequently, miRNA expression profiles from 2 independent validation data sets, GSE23739 (40 GC tissues and 40 adjacent normal mucosae; validation data set 1) and GSE33743 (37 GC tissues and 4 adjacent normal mucosae; validation data set 2), were evaluated for the diagnostic performance of the discovered miRNA candidates. The data are accessible at the NCBI Genetic Expression Omnibus database.^16^

In the tissue validation phase, quantitative reverse-transcription–polymerase chain reaction (qRT-PCR) assays were performed to interrogate the expression levels of candidate miRNAs in 50 pairs of matched, fresh-frozen, primary tumor and adjacent normal tissues from patients with GC. These specimens were obtained from patients enrolled at Kumamoto University during 2014 and 2015.

In the retrospective serum validation phase, 216 serum specimens were collected from patients with GC and 43 specimens from endoscopically negative patients enrolled at Kumamoto University between 2010 and 2015 for internal validation, and 288 serum specimens were collected from patients with GC and 39 specimens from healthy participants enrolled at Nagoya University between 1997 and 2015 for external validation. Using circulating miRNA expression profiles for all 115 patients with GC and a matched number of healthy participants (2759 randomly selected from a larger cohort), a 3-miRNA signature was developed using logistic regression and elastic net regularization, followed by establishment of a risk probability model.

In the prospective serum validation phase, serum specimens were collected from 176 patients with GC and 173 healthy participants, matched by age and sex, who were prospectively recruited from March 2017 to August 2018 at Jiangsu University (Table).

**Table.** Demographics of Patients and Healthy Participants

Characteristic	Participants, No. (%)					
	Tissue cohort		Retrospective serum cohorts			Prospective serum cohort (validation) (n = 349)
	Discovery (n = 598)^a^	Validation (n = 100)	Kumamoto (n = 259)	Nagoya (n = 327)	Training (n = 2874)	
**Diagnosed GC**						
Total	513 (85.8)	50 (50)	216 (83.4)	288 (88.1)	115 (4.0)	176 (50.4)
Sex						
Men	284 (55.4)	38 (76)	137 (63.4)	211 (73.3)	NR	124 (70.5)
Women	158 (30.8)	12 (24)	79 (36.6)	77 (26.7)	NR	52 (29.5)
Age, mean (SE), y	65.7 (0.5)	67.6 (1.7)	65.9 (1.0)^b^	66.1 (0.7)	NR	66 (0.7)
Cancer stage						
I	58 (11.3)	6 (12)	84 (38.9)	133 (46.2)	NR	54 (30.7)
II	131 (25.5)	18 (36)	33 (15.3)	40 (13.9)	NR	18 (11.4)
III	188 (36.7)	19 (38)	32 (14.8)	62 (21.5)	NR	87 (48.9)
IV	46 (9.0)	7 (14)	47 (21.7)	52 (18.0)	NR	NR
Unstaged	90 (17.5)	NR	20 (9.3)	1 (0.4)	NR	17 (9.0)
Serum CEA, mean (SE), ng/mL	NR	5.92 (1.68)^c^	6.98 (2.51) (n = 147)^c^	16.1 (7.77 (n = 235)^d^	NR	4.26 (0.71) (n = 94)^d^
Serum CA19-9, mean (SE), U/mL	NR	37.8 (17.1)^b^	30.9 (10.4) (n = 145)^b^	87.8 (24.5) (n = 229)^b^	NR	15.3 (4.33) (n = 96)^b^
Noncancerous gastric mucosa	85 (14.2)	50 (50)	NR	NR	NR	NR
**Healthy participants**						
Total	NR	NR	NR	39 (11.9)	2759 (96.0)^e^	173 (49.6)
Sex						
Men	NR	NR	NR	20 (51.3)	NR	111 (64.2)
Women	NR	NR	NR	19 (48.7)	NR	62 (35.8)
Age, mean (SE), y	NR	NR	NR	39.0 (1.76)	NR	70 (1.01)
Serum CEA, mean (SE), ng/mL	NR	NR	NR	NR	NR	2.13 (0.10) (n = 61)^d^
Serum CA19-9, mean (SE), U/mL^a^	NR	NR	NR	NR	NR	11.8 (0.66) (n = 59)^b^
**Endoscopically negative**						
Total	NR	NR	43 (16.6)	NR	NR	NR
Sex						
Men	NR	NR	24 (55.8)	NR	NR	NR
Women	NR	NR	19 (44.2)	NR	NR	NR
Age, mean (SE), y	NR	NR	59.5 (2.21)	NR	NR	NR
Serum CEA, mean (SE), ng/mL	NR	NR	6.48 (6.50) (n = 22)^c^	NR	NR	NR
Serum CA19-9, mean (SE), U/mL	NR	NR	21.5 (28.8) (n = 19)^b^	NR	NR	NR

Abbreviations: CA19-9, cancer antigen 19-9; CEA, carcinoembryonic antigen; SE, standard error.

SI conversion factor: To convert CEA to kilounits per liter, multiple by 1.0.

^a^ Discovery cohorts include TCGA (n = 477), GSE23739 (n = 80), and GSE33743 (n = 41).

^b^ Cutoff value is 3.4 ng/mL.

^c^ Cutoff value is 5.0 ng/mL.

^d^ Cutoff value is 37 U/mL.

^e^ 115 healthy participants were randomly selected from 2759 healthy participants for analysis.

### Genome-Wide miRNA Data Analysis

In the discovery phase, we first analyzed genome-wide miRNA sequencing data from TCGA data downloaded from the Firehose Broad GDAC portal^17^ (ie, the discovery cohort) to identify candidate miRNAs for the early detection of patients with GC. The miRNA expression levels were first log_2_ transformed. Differential miRNA expression analysis was performed between GC and adjacent normal tissues using the bioconductor package and limma package in R.^18^ Area under the curve (AUC) was used to evaluate the predictive power of each miRNA candidate’s expression level in distinguishing GC from normal tissue. For the selection of the initial miRNA target genes from the discovery cohort, we used the following: criteria Benjamini Hochberg-adjusted *P* < 1 × 10^−5^, absolute log_2_-fold change greater than 1,^18^ AUC greater than 0.9 and upregulated in GC. Two additional miRNAs, miR-196a-2 and miR-18a, were also included in the initial target genes because of their high discriminative power (AUC, 0.90 and 0.87, respectively), and because they were highly overexpressed in GC patients (log_2_-fold change greater than 2, Benjamini Hochberg-adjusted *P* < 1 × 10^−5^).

### Statistical Analyses

All statistical analyses were performed using Medcalc version 12.3.0 (Medcalc Software bvba), GraphPad Prism version 5.0 (GraphPad Software), and R version 3.3.3 (R Project for Statistical Computing). Differential miRNA expression analysis was performed using the limma package in R, and the resulting *P* values were adjusted using the Benjamini-Hochberg method. The Wilcoxon signed-rank test, the Mann-Whitney U test, and the Kruskal-Wallis test were used to analyze miRNA expression data obtained from qRT-PCR experiments, and results were expressed as mean with standard errors (SE). Silhouette width was calculated using R package cluster with Euclidean distance. Pearson correlation coefficient was calculated by R package stats and graphically displayed by R package corrplot. The principal components analysis and other statistical analyses including logistic regression were also performed using the stats package in R. AUCs derived from receiver operating characteristic (ROC) curves were calculated with CIs using the pROC package in R; ROC curves were compared using DeLong tests in the pROC package. The 10-miRNA random forest classifier was trained using the caret package in R. Logistic regression with elastic net regularization was performed using the glmnet package through the caret package in R with default configuration of optimization. ORs were calculated using the vcd package in R. Detailed materials and methods are described in the Supplement. The threshold for significance was *P* < .05 in 2-sided tests (except for Delong test and boxplot analyses, which used 1-sided tests).

## Results

### Ten-miRNA Signature for GC Diagnosis

**Figure 1.**
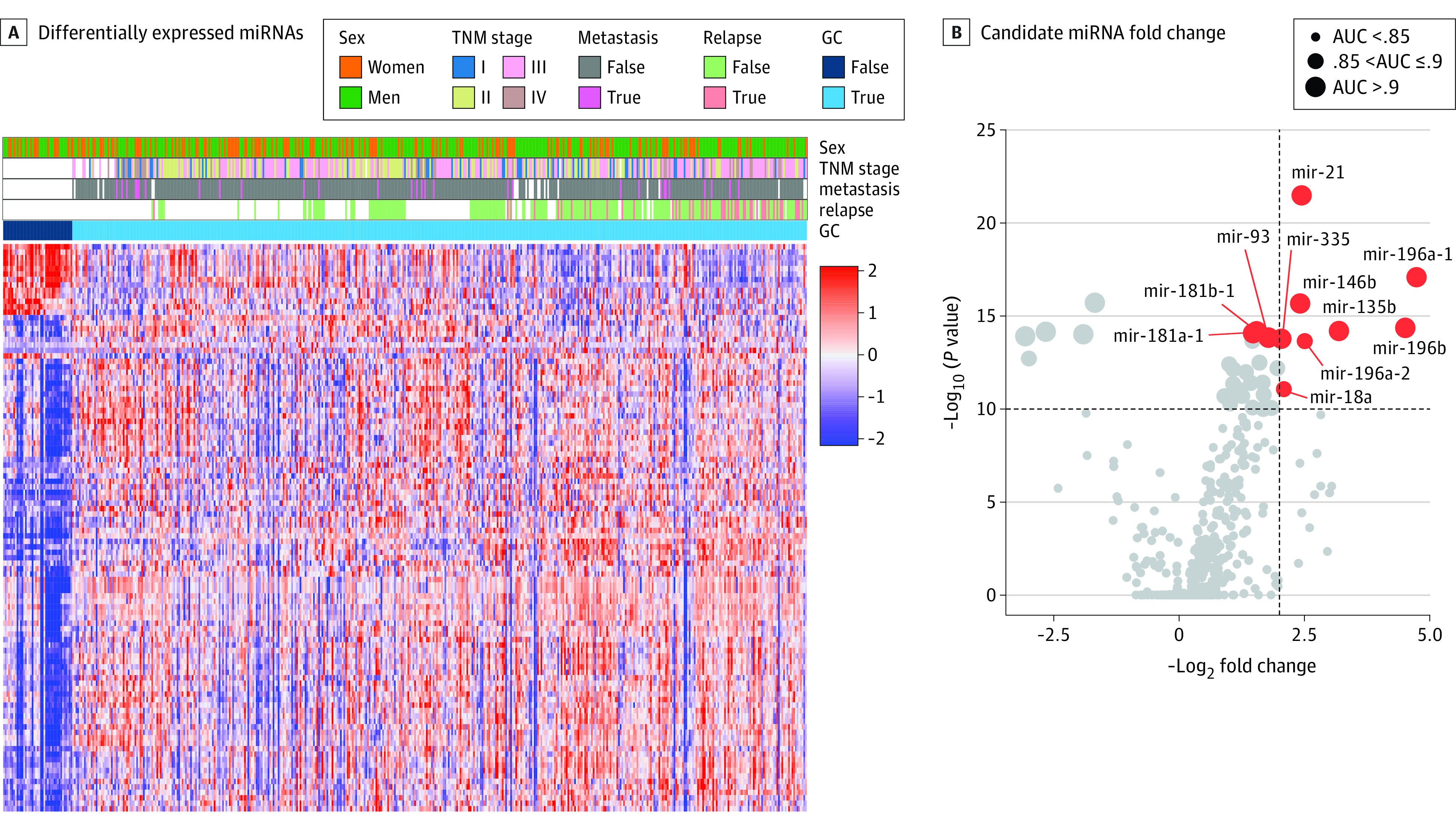
Genome-Wide Discovery of miRNA Candidates for GC Diagnosis Panel A represents a heat map of all differentially expressed microRNAs (miRNAs) in The Cancer Genome Atlas gastric cancer (GC) data set (Benjamini-Hochberg–adjusted *P* < .05 and absolute fold change greater than 1; 104 miRNA candidates). Samples included from 436 patients with GC and 41 normal adjacent tissue samples. B, The 2-dimensional plot illustrates the 11 candidate miRNAs (with area under the curve [AUC] greater than 0.85 and absolute fold change greater than 2, or AUC greater than 0.9).

A total of 598 total patient samples were included in the genome-wide expression profiling analysis stage (284 [55.4%] from men; mean [SE] patient age, 65.7 [0.5] years); 586 were included in the 2 retrospective GC serum cohorts (348 [59.4%] men; mean [SE] age, 66.0 [0.7] years). Demographic and clinicopathological characteristics for all cohorts examined in our study are shown in the Table. We employed an unbiased genome-wide biomarker discovery approach to identify a clinically relevant circulating miRNA signature for the early detection of GC in patients. From a discovery data set, we identified 10 miRNAs (miR-21, miR-196a, miR-146b, miR-196b, miR-135b, miR-18a, miR-181b, miR-181a, miR-93, and miR-335) (Figure 1). To examine the stability and consistency of this 10-miRNA combination within GC and normal groups, we conducted a silhouette analysis. The mean (SD) silhouette score improved from 0.21 (0.21) (calculated by Euclidean distance using all differentially expressed miRNAs) to 0.4 (0.21) (using the 10 selected miRNAs, eFigure 2 in the Supplement). To verify the predictive performance of the selected miRNAs for diagnosing GC, we next performed principal component analysis in the TCGA data set and 2 additional independent data sets, validation data sets 1 (GSE23739) and 2 (GSE33743). Principal component analysis showed that the 10 miRNAs discriminated between GC and normal samples (eFigure 2 in the Supplement).

**Figure 2.**
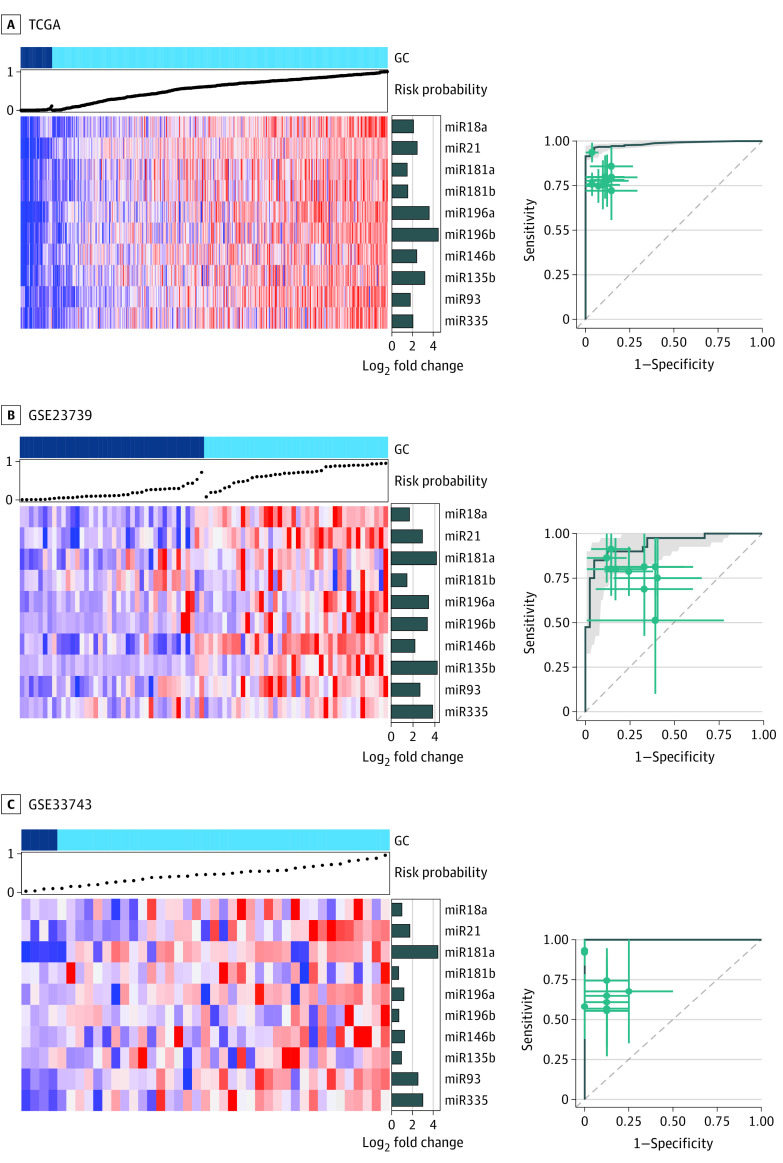
Establishment and Validation of a 10-miRNA–Based Signature in Public Cohorts Heat maps of expression (left) and receiving operating characteristic (ROC) curves (right) are shown for the 10 candidate microRNAs (miRNAs) in the 3 cohorts. Predictive probabilities for heatmaps were calculated using the random forest classifier model, shown in the top panel. ROC curves are shown with 95% CIs. Green lines in right panel indicate 95% CIs of sensitivity and specificity for each miRNA; green points, best threshold for sensitivity and specificity.

To evaluate the performance of these miRNA biomarkers in combination, we trained a random forest classifier using miRNA expression levels of the 10 miRNAs for GC and matched adjacent normal tissues in the discovery data set. The predictive probabilities calculated using the random forest classifier model demonstrated a robust diagnostic value in the entire discovery data set (AUC, 0.99; 95% CI, 0.98-1.00) and the 2 validation data sets (AUC, 0.94; 95% CI, 0.89-0.99; and AUC, 1.00; 95% CI, 1.00-1.00, respectively) (Figure 2).

To better appreciate the functional relevance of our candidate miRNAs, we constructed a miRNA-mRNA regulatory network based on experimentally validated miRNA-target interactions, as reported in the miRTarBase database version 8 (National Chiao Tung University).^19^ In total, we identified 322 genes (log_2_-fold change > 2 and Benjamini-Hochberg–adjusted *P* < .05) that were differentially expressed between GC and normal samples and were direct downstream targets of these miRNAs (eFigure 3, eTable 2 in the Supplement). As expected, the miRNA target genes were significantly enriched in cancer-related signaling pathways, as well as in previously reported gene expression signatures that discriminated between GC and normal tissues, highlighting their functional significance (eFigure 3, eTable 3 in the Supplement).^20^

Next, to further validate the 10 identified miRNA candidates and eliminate the miRNAs that may discriminate other background characteristics including age, gender, and race that are not equally distributed between the tumor and subset of patients who also had adjacent normal tissue available, we assessed the expression of these miRNAs in 50 matched pairs of fresh-frozen GC tissues and adjacent normal mucosae using qRT-PCR assays. Compared with normal tissues, 9 of the 10 miRNAs were confirmed to be overexpressed in GC tissue (eFigure 4, eTable 4 in the Supplement), highlighting the specificity of these biomarkers for GC, and were selected for subsequent analyses.

### Identification of Patients With GC

To validate and explore the feasibility of translating the tissue-based biomarkers into a circulating miRNA-based signature for the noninvasive diagnosis of GC, we first evaluated the expression levels of the 9 miRNAs in an in-house serum internal validation cohort (Kumamoto cohort GC: 21 patients with GC and 43 endoscopically negative patients). Using the endoscopically negative patients allowed us to acquire more purified results because randomly collected healthy participants have potential risk to include patients who have unproven GCs. Eight out of the 9 miRNA biomarkers were significantly upregulated in sera from patients with GC vs healthy participants (eFigure 4 in the Supplement). To further optimize this biomarker signature, we filtered out 2 miRNAs that exhibited very low expression in serum (miR-196a and miR-196b, raw CT >35) and 2 other miRNAs that were highly correlated with other miRNAs (miR-21: average correlation, 0.65; miR-181a: average correlation, 0.66) (eFigure 5 in the Supplement), yielding a signature of 5 miRNAs: miR-18a, miR-93, miR-146b, miR-181b, and miR-335. We subsequently evaluated the predictive performance of each of these miRNAs in the Kumamoto cohort by calculating the AUC, odds ratio (OR), specificity, sensitivity, and accuracy based on Youden’s index (eTable 5 in the Supplement).

**Figure 3.**
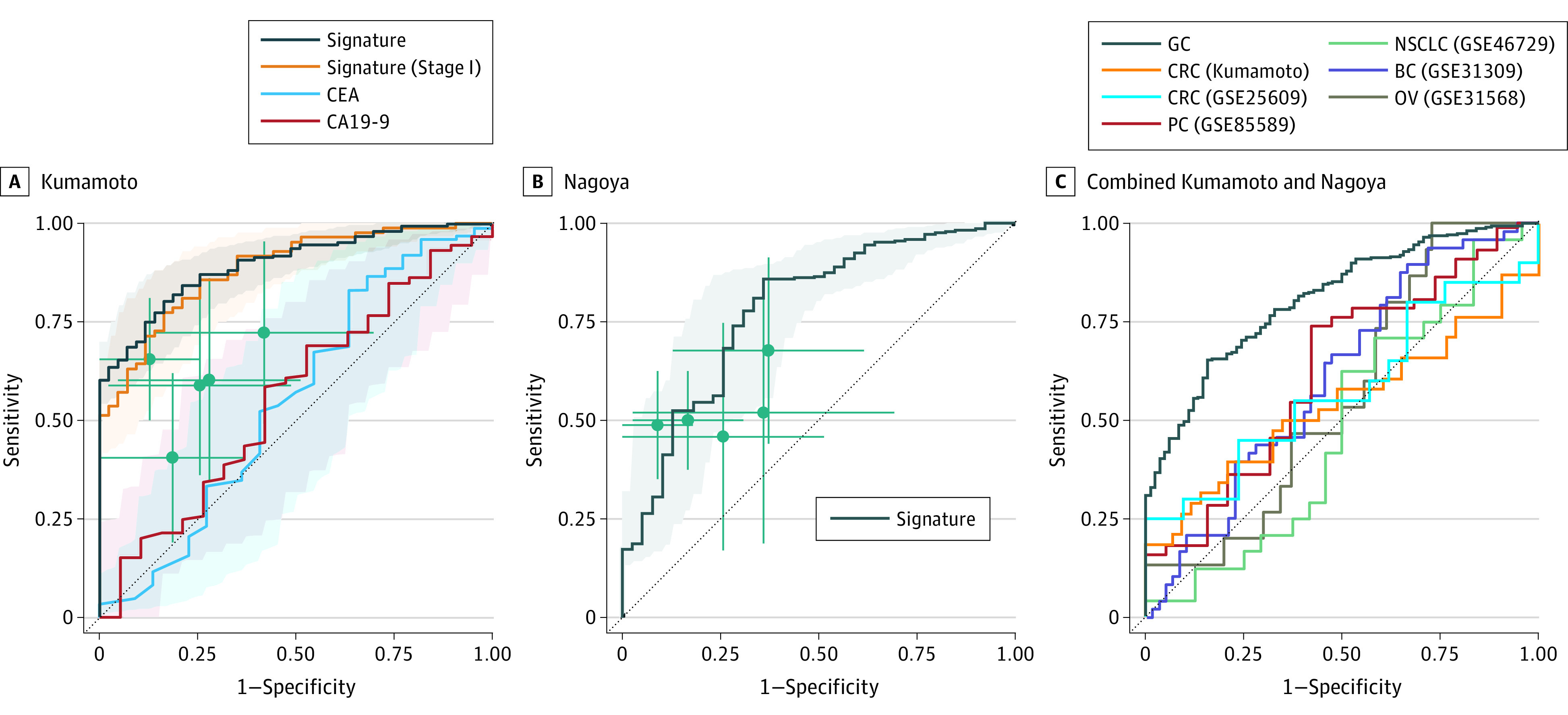
Establishment and Validation of a Circulating miRNA Signature for Noninvasive Detection of GC Risk scores were calculated based on 5-fold cross-validation for 100 times. Receiver operating characteristic (ROC) curves are shown with 95% CI. Green lines represent 95% CIs of sensitivity and specificity for each microRNA (miRNA); green points, the best threshold for sensitivity and specificity. BC indicates bladder cancer; CA19-9, cancer antigen; CEA, carcinoembryonic antigen; CRC, colorectal cancer; GC, gastric cancer; NSCLC, non–small cell lung cancer; OV, ovarian cancer; PC, pancreatic cancer.

Because individual miRNAs may have variable diagnostic value, signature-based biomarker approaches have substantially improved efficacy. Following a 5-fold cross-validation (ie, 100 times) in the Kumamoto cohort, we performed multivariate logistic regression on 80% of the patient cohort (using random selection) and used the derived model to calculate risk scores in the remaining 20% of the cohort. We used the mean risk scores calculated from the cross-validation analyses to determine the performance of the circulating 5-miRNA signature, which demonstrated high diagnostic potential for the identification of patients with GC (AUC, 0.90; 95% CI, 0.85-0.94) (Figure 3, eFigure 5 and eTable 6 in the Supplement). We then evaluated the predictive performance of the circulating miRNA signature in the Kumamoto cohort by calculating the AUC curve, accuracy, sensitivity, specificity, positive predictive value (PPV), and negative predictive value (NPV) based on Youden’s index (eFigure 5 and eTable 7 in the Supplement). Our subset analysis of stage I cancer patients within this cohort revealed that this miRNA signature panel maintained its significantly high diagnostic performance in the serum specimens of these early-stage patients (AUC, 0.89; 95% CI, 0.83-0.94) (Figure 3, eTable 7 in the Supplement). Furthermore, when we compared patients at various stages of GC with healthy participants, this miRNA signature exhibited a consistent and high diagnostic performance (risk probability [RP]: healthy participants, 0.558; stage I, 0.877; stage II, 0.903; stage III, 0.868; stage IV, 0.893; *P* < .0001 in 1-sided Kruskal-Wallis tests) (eFigure 5 in the Supplement).

### Five-miRNA Signature as an Independent Predictor of GC

To further evaluate the accuracy of our circulating 5-miRNA signature compared with other clinical factors, we performed univariate and multivariate analyses, which revealed that our miRNA biomarker signature had superior diagnostic power vs other clinical features, including age, sex, and the 2 most commonly used GC tumor markers, CEA and CA19-9 (eTable 6 in the Supplement). Accordingly, using the Kumamoto cohort, we evaluated the ability of a combination signature to provide superior diagnostic performance in the identification of patients with GC. Specifically, the circulating miRNA signature demonstrated significant superiority for detecting GC compared with CEA (AUC, 0.55; 95% CI, 0.40-0.70; *P* < .01 in DeLong tests) and CA19-9 (AUC, 0.56; 95% CI, 0.42-0.70; *P* < .01 in DeLong tests) (Figure 3; and eTable 7 in the Supplement).

To further validate the robustness of our circulating miRNA signature, we analyzed qRT-PCR results from another independent, external serum cohort (ie, the Nagoya cohort), which included data from 288 patients with GC and 39 healthy participants. In support of our previous findings, using the risk score formula derived from the Kumamoto cohort, our miRNA signature demonstrated high diagnostic potential in this independent validation cohort (AUC, 0.78; 95% CI, 0.70-0.86) (Figure 3; eFigure 5 in the Supplement).

### Significant Specificity of 5-miRNA Signature for Diagnosing GC

To evaluate the specificity of our 5-miRNA signature, we compared its performance on GC with other gastrointestinal cancers (eg, colorectal cancer, pancreatic cancer) and other representative malignant neoplasms (non–small cell lung cancer, breast cancer, ovarian cancer) using in-house and public serum data sets. As a result, our miRNA signature showed significantly higher AUC on GC (in the combined Kumamoto and Nagoya cohorts) than other cancers (GC, 0.81; colorectal cancer [Kumamoto], 0.54; colorectal cancer [GSE25609], 0.58; pancreatic cancer, 0.63; non–small cell lung cancer, 0.48; breast cancer, 0.61; ovarian cancer, 0.56) (Figure 3; eTable 7 in the Supplement). For instance, the miRNA signature had significantly poorer performance on distinguishing patients with colorectal cancer from endoscopically negative patients (Kumamoto cohort: AUC, 0.54; 95% CI, 0.41-0.67) and from healthy controls (GSE25609 cohort: AUC, 0.58; 95% CI, 0.39-0.76). Collectively, these data indicate that our miRNA signature is most robust and specific for the identification of patients with GC.

To further confirm the cancer specificity and the fact that our circulating miRNA candidates were derived from cancer tissue, we examined their performance in 22 pairs of preoperative and postoperative serum specimens from patients with GC. If the candidate miRNAs were derived from GC, we expect reduced levels of candidate miRNAs in the circulation following the removal of the tumor. We observed that expression of the circulating miRNA signature and the corresponding risk probability plummeted 30 days after curative surgery (preoperation, 0.761; postoperation, 0.414; *P* < .001) (eFigure 5 in the Supplement); this supports the diagnostic relevance of our biomarker signature and suggests that the elevated expression of these miRNAs in presurgical serum specimens is because of the primary GC.

### Performance of 3-miRNA Signature vs Conventional Markers in a Prospective Cohort of Patients with GC

**Figure 4.**
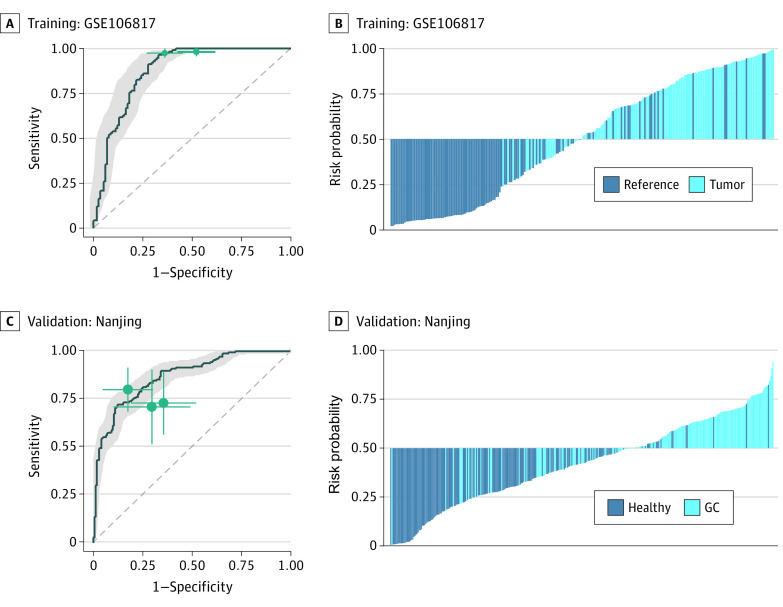
Establishment of a 3-Circulating–miRNA Signature and Evaluation in a Prospective Validation Serum Cohort The multivariate logistic regression model was trained based on 155 patients with gastric cancer (GC) and 155 noncancer controls. Receiver operating characteristic curves are shown with 95% CI. Green lines indicate 95% CIs of sensitivity and specificity for each microRNA (miRNA); green points, the best threshold for sensitivity and specificity. C and D, The multivariate logistic regression model was validated in a prospective validation cohort (Nanjing).

To further evaluate cross-platform robustness and optimize a cost-efficient assay that includes the minimally effective number of biomarkers, we analyzed individual circulating miRNA expression profiles in sera from 115 patients with GC and an equal number of healthy participants (GSE106817 training cohort). We confirmed that all 5 miRNAs were significantly upregulated in patients with GC compared with healthy participants (eFigure 6 in the Supplement). Using logistic regression with elastic net regularization and bootstrapping (α= 0.55 and λ = 0.052, optimized by 25 bootstrapped resampling), we successfully narrowed down our signature to 3 biomarkers (miR-18a, miR-181b, and miR-335), which offered the highest diagnostic accuracy without redundancy. We used a multivariate logistic regression model to derive a risk scoring formula: logit(*P*) = (−0.2941 + (0.5042 × miR-18a) + (0.1351 × miR-181b) + (0.1130 × miR-335)). Risk probabilities calculated using this formula demonstrated the effective diagnostic performance of this 3-miRNA signature (AUC, 0.87; 95% CI, 0.83-0.92; OR, 56.23; 95% CI, 19.28-164.01) (Figure 4).

To demonstrate the clinical applicability of the optimal 3-miRNA signature, we prospectively recruited and collected sera from 176 patients with GC and 173 healthy participants (ie, the Nanjing cohort). Using qRT-PCR data for the 3 miRNAs, we calculated risk probabilities using the established scoring formula, which showed that our 3-miRNA signature performed robustly in the identification of patients with GC (AUC, 0.86; 95% CI, 0.83-0.90; sensitivity, 71.6%; specificity, 87.9%) (Figure 4; and eFigure 7 in the Supplement). Compared with the conventional tumor markers CEA and CA19-9, our miRNA signature consistently demonstrated superior diagnostic performance for all-stage GC (AUC: signature, 0.86; CEA, 0.65; CA19-9, 0.67; *P* < .001 in DeLong tests) (eTable 8, eFigure 7 in the Supplement). More importantly, our 3-miRNA signature discriminated between patients with stage I GC and healthy participants (AUC, 0.85; 95% CI, 0.79-0.91; sensitivity, 72%; specificity, 88%) (eTable 8, eFigure 7 in the Supplement). Furthermore, compared with healthy participants, we observed significantly elevated risk probabilities for patients in any of the 4 stages of GC and those with a precancerous lesion (RP: healthy, 0.285; precancerous, 0.510; stage I, 0.539; stage II, 0.581; stage III, 0.548; stage IV, 0.602; *P* < .001 in 1-sided *t* tests) (eFigure 7). Univariate and multivariate analyses confirmed that our circulating 3-miRNA signature emerged as the most significant predictor for detecting GC (eTable 9 in the Supplement).

Using the sensitivity and specificity calculated in the validation cohort, we performed a cost-effectiveness (CE) analysis to evaluate the effectiveness of our miRNA signature for large-scale screening. Using a Markov model–based CE analysis, we estimated that a large-scale screening using our miRNA signature was significantly more cost-effective relative to the current clinical practice of endoscopic screening (incremental cost-effectiveness ratio, CNY ¥16162.5 per quality-adjusted life-year [USD $2304.80 per quality-adjusted life-year]) (eTable 10 in the Supplement). Taken together, this multicenter cohort study showed that our circulating miRNA signature provides a cost-efficient, robust assay for noninvasive early detection of GC, with promise for future clinical translation.

## Discussion

Currently, screening for GC is recognized as the most effective strategy for reducing GC-associated mortality. Although endoscopic surveillance is regarded as the criterion standard for GC screening in many of Asian countries, because of its invasiveness and the cost, it is not an ideal screening method.^21^ Therefore, there is a need for noninvasive approaches, including liquid biopsy-based molecular biomarker assays. In this study, we developed and validated a circulating miRNA signature in retrospective cohorts and demonstrated the robustness of the miRNA signature against conventional tumor markers. Subsequently, we evaluated the performance in a large prospective cohort. In addition, we showed that our circulating miRNA signature could be more cost-effective than the endoscopic screening.

Advancements in high-throughput technologies and the availability of large multiomic and clinical data sets have permitted comprehensive molecular characterization of cancer, including for patients with GC.^22^ Due to their inherent stability and high abundance, miRNAs are regarded as one of the most attractive liquid biopsy-based biomarker substrates.^23^ Despite the potential associated with using circulating miRNAs for detection of GC, the robustness of individual miRNAs is quite limited for GC diagnosis.^12,13,14,15^ Therefore, we used a comprehensive and unbiased biomarker discovery approach, which included analyses of several hundred patients from multiple centers and followed this initial discovery step with validation in independent data sets. Consequently, all of the miRNAs prioritized and validated in our final signature are associated with oncogenic pathways that have previously been reported to play a pathogenic role in GC.^24,25,26,27^ The observation that the miRNAs in our signature were consistently overexpressed in multiple independent GC data sets further validates their clinical importance and supports their inclusion in this diagnostic biomarker signature.

### Limitations

There are several limitations to this study. We deliberately prioritized miRNA biomarkers that were overexpressed in GC tissues with the hypothesis that such miRNAs are the most likely to be released into systemic circulation. However, recent studies have shown that some miRNAs that do not accumulate in tissues may still be excreted in extracellular-vesicles such as exosomes.^28^ In addition, we plan to conduct a large prospectively recruited study that includes the patients who are at risk of having GC and healthy participants to further validate our miRNA signature.

## Conclusions

This study established a robust, noninvasive, circulating miRNA signature for GC detection, and validated its diagnostic potential in multiple independent patient cohorts, both retrospective and prospective, highlighting its potential application for the early detection of patients with GC.

## Notes

Supplement.
eMethods.
**eFigure 1.** Study Design for the Identification of a Circulating miRNA Expression Signature for Early Detection of GC **eFigure 2.** Genome-wide Discovery of miRNA Candidates for GC Diagnosis **eFigure 3.** miRNA Regulatory Network Analysis and Functional Analysis of the miRNA Target Genes **eFigure 4.** Tissue Validation and Serum Validation for the 10 miRNA Candidates **eFigure 5.** Validation of the 5 Circulating miRNAs in the Kumamoto Cohort **eFigure 6.** Validation of the 5 Circulating miRNAs in a Public Serum Cohort (GSE106817) **eFigure 7.** Establishment of a 3-Circulating-miRNA Signature and Evaluation in a Prospective Validation Serum Cohort **eTable 1.** Statistical Summary of the Initially Identified 11 miRNAs from the Discovery Dataset **eTable 2.** MiRNA-mRNA Interactions in the Regulatory Network **eTable 3.** Functional Analysis of miRNA Target Genes Identified 61 Significantly Enriched Functional Gene Sets (BH-adjusted *P* Value < 0.0001) **eTable 4.** Comparison Between the Expression of the 10 miRNAs in GC and Normal Frozen Tissues **eTable 5.** Statistical Evaluation of the Diagnostic Value of the miRNAs in Differentiating GC Patients from Healthy Participants in the Serum Internal Validation Cohort **eTable 6.** Univariate and Multivariate Analysis Using Clinical Factors and the Circulating miRNA Signature **eTable 7.** Analyses Comparing Our 5-Circulating-miRNA Signature With CEA and CA19-9 for Non-Invasive Detection of Gastric Cancer (GC) Across All Stages and Stage I in the Prospective Validation Cohort **eTable 8.** Comparison of the Performance of Our 3-Circulating-miRNA Signature Against CEA and CA19-9 for Non-Invasive Detection of All-Stage GC and Stage I GC in the Prospective Validation Cohort **eTable 9.** Univariate and Multivariate Analyses Comparing Our 3-Circulating-miRNA Signature With Age, Sex, CEA, and CA19-9 for Non-Invasive Detection of GC Across All Stages and Stage I in the Prospective Validation Cohort **eTable 10.** Results of Cost-Effectiveness Analysis **eTable 11.** Clinical Assumptions of the Hypothetical Cohort Used for the Cost Effectiveness Analysis
