# Supplementary material for: Assessment of the Diagnostic Efficiency of a Liquid Biopsy Assay for Early Detection of Gastric Cancer
Source: JAMA Netw Open. 2021 Aug 24;4(8):e2121129. doi: 10.1001/jamanetworkopen.2021.21129 (PMC8385601; doi:10.1001/jamanetworkopen.2021.21129)
Supplement: Supplement. — eMethods. eFigure 1. Study Design for the Identification of a Circulating miRNA Expression Signature for Early Detection of GC eFigure 2. Genome-wide Discovery of miRNA Candidates for GC Diagnosis eFigure 3. miRNA Regulatory Network Analysis and Functional Analysis of the miRNA Target Genes eFigure 4. Tissue Validation and Serum Validation for the 10 miRNA Candidates eFigure 5. Validation of the 5 Circulating miRNAs in the Kumamoto Cohort eFigure 6. Validation of the 5 Circulating miRNAs in a Public Serum Cohort (GSE106817) eFigure 7. Establishment of a 3-Circulating-miRNA Signature and Evaluation in a Prospective Validation Serum Cohort eTable 1. Statistical Summary of the Initially Identified 11 miRNAs from the Discovery Dataset eTable 2. MiRNA-mRNA Interactions in the Regulatory Network eTable 3. Functional Analysis of miRNA Target Genes Identified 61 Significantly Enriched Functional Gene Sets (BH-adjusted P Value < 0.0001) eTable 4. Comparison Between the Expression of the 10 miRNAs in GC and Normal Frozen Tissues eTable 5. Statistical Evaluation of the Diagnostic Value of the miRNAs in Differentiating GC Patients from Healthy Participants in the Serum Internal Validation Cohort eTable 6. Univariate and Multivariate Analysis Using Clinical Factors and the Circulating miRNA Signature eTable 7. Analyses Comparing Our 5-Circulating-miRNA Signature With CEA and CA19-9 for Non-Invasive Detection of Gastric Cancer (GC) Across All Stages and Stage I in the Prospective Validation Cohort eTable 8. Comparison of the Performance of Our 3-Circulating-miRNA Signature Against CEA and CA19-9 for Non-Invasive Detection of All-Stage GC and Stage I GC in the Prospective Validation Cohort eTable 9. Univariate and Multivariate Analyses Comparing Our 3-Circulating-miRNA Signature With Age, Sex, CEA, and CA19-9 for Non-Invasive Detection of GC Across All Stages and Stage I in the Prospective Validation Cohort eTable 10. Results of Cost-Effectiveness Analysis eTable 11. Clinical Assu [file jamanetwopen-e2121129-s001.pdf]

Supplemental Online Content

Izumi D, Zhu Z, Chen Y, et al. Assessment of the diagnostic efficiency of a liquid biopsy assay for early detection of gastric cancer. *JAMA Netw Open.* 2021;4(8):e2121129. doi:10.1001/jamanetworkopen.2021.21129

eMethods.

eFigure 1. Study Design for the Identification of a Circulating miRNA Expression Signature for Early Detection of GC

eFigure 2. Genome-wide Discovery of miRNA Candidates for GC Diagnosis

eFigure 3. miRNA Regulatory Network Analysis and Functional Analysis of the miRNA Target Genes

eFigure 4. Tissue Validation and Serum Validation for the 10 miRNA Candidates

eFigure 5. Validation of the 5 Circulating miRNAs in the Kumamoto Cohort

eFigure 6. Validation of the 5 Circulating miRNAs in a Public Serum Cohort (GSE106817)

eFigure 7. Establishment of a 3-Circulating-miRNA Signature and Evaluation in a Prospective Validation Serum Cohort

eTable 1. Statistical Summary of the Initially Identified 11 miRNAs from the Discovery Dataset

eTable 2. MiRNA-mRNA Interactions in the Regulatory Network

eTable 3. Functional Analysis of miRNA Target Genes Identified 61 Significantly Enriched Functional Gene Sets (BH-adjusted *P* Value < 0.0001)

eTable 4. Comparison Between the Expression of the 10 miRNAs in GC and Normal Frozen Tissues

eTable 5. Statistical Evaluation of the Diagnostic Value of the miRNAs in Differentiating GC Patients from Healthy Participants in the Serum Internal Validation Cohort

eTable 6. Univariate and Multivariate Analysis Using Clinical Factors and the Circulating miRNA Signature

eTable 7. Analyses Comparing Our 5-Circulating-miRNA Signature With CEA and CA19-9 for Non-Invasive Detection of Gastric Cancer (GC) Across All Stages and Stage I in the Prospective Validation Cohort

© 2021 Izumi D et al. *JAMA Network Open.*

eTable 8. Comparison of the Performance of Our 3-Circulating-miRNA Signature Against CEA and CA19-9 for Non-Invasive Detection of All-Stage GC and Stage I GC in the Prospective Validation Cohort

eTable 9. Univariate and Multivariate Analyses Comparing Our 3-Circulating-miRNA Signature With Age, Sex, CEA, and CA19-9 for Non-Invasive Detection of GC Across All Stages and Stage I in the Prospective Validation Cohort

eTable 10. Results of Cost-Effectiveness Analysis

eTable 11. Clinical Assumptions of the Hypothetical Cohort Used for the Cost Effectiveness Analysis

This supplemental material has been provided by the authors to give readers additional information about their work.

© 2021 Izumi D et al. *JAMA Network Open.*

eMethods

Study design and participants

We analyzed more than 1900 tissue and serum specimens from patients with GC, adjacent normal tissues and healthy participants in a four-phase study, which involved a biomarker discovery phase, a tissue validation phase, a retrospective serum validation phase, and a prospective serum performance evaluation phase (**Supplementary Figure 1**).

The biomarker discovery cohort (436 GC tissues and 41 adjacent normal mucosae from TCGA, consisting of small RNA-sequencing data and generated by the TCGA Research Network: https://www.cancer.gov/tcga) was analyzed to identify miRNA candidates for the early diagnosis of GC. Subsequently, miRNA expression profiles from two independent validation datasets, GSE23739 (40 GC tissues and 40 adjacent normal mucosae; validation dataset 1) and GSE33743 (37 GC tissues and 4 adjacent normal mucosae; validation dataset 2), were evaluated for the diagnostic performance of the discovered miRNA candidates. The data are accessible at NCBI GEO database (*32*).

During the tissue validation phase, qRT-PCR assays were performed to interrogate the expression levels of candidate miRNAs in 50 pairs of matched, fresh-frozen, primary tumor and adjacent normal tissues from patients with GC. These specimens were obtained from patients enrolled at Kumamoto University, Japan, who underwent curative resection without any pretreatment during 2014–2015. The matched corresponding normal tissues were collected from the greater curvature, and at least 5 cm away from the cancer tissue.

In the retrospective serum validation phase, two independent patient cohorts were analyzed, totaling 586 serum specimens. In the serum internal validation cohort, 216 serum specimens were collected from patients with GC and 43 specimens from endoscopically negative patients enrolled at Kumamoto University, Japan, between 2010 and 2015. Endoscopically negative patients were those who had esophagogastroduodenoscopy and diagnosed as negative for gastric cancer and had never diagnosed as any other cancer recruited at Kumamoto University. In the serum external validation

© 2021 Izumi D et al. *JAMA Network Open.*

cohort, sera were collected from 288 patients with GC and 39 serum specimens from healthy participants enrolled at Nagoya University, Japan, between 1997 and 2015. Healthy participants were randomly recruited at Nagoya University from people who have no diagnosed disease. In addition, the status of tumor markers, CEA and CA19-9, were also collected from the medical records of each institution. Written informed consent was obtained from all patients, and the institutional review boards of all participating institutions approved the study. In the serum validation phase, the three independent patient cohorts (TCGA, GSE23739, GSE33743) were analyzed again. Based on qRT-PCR data in serum, two miRNAs with low expression (miR-196a and miR-196b) and two others that were highly correlated with one another (miR-21 and miR-181a) were excluded from subsequent signature construction. The diagnostic potential of the initial signature was evaluated by performing a 5-fold cross-validation in our serum cohort (Kumamoto, n = 259) for internal validation, as well as an independent external validation cohort (Nagoya, n = 327). To further optimize a robust risk formula, a public dataset (GSE106817 cohort, data accessible at NCBI GEO database (*33*)) was used for miRNA expression profiling in sera from patients with GC and healthy participants. Using circulating miRNA expression profiles for all 115 patients with GC and a matched number of healthy participants (randomly selected from a larger cohort [n = 2759]), a 3-miRNA signature was developed using logistic regression and elastic net regularization, followed by establishment of a risk probability model. Public serum datasets were downloaded from GEO: GSE25609 (colorectal cancer, CRC), GSE85589 (pancreatic cancer, PC), GSE46729 (non-small cell lung cancer, NSCLC), GSE31309 (breast cancer, BC), and GSE31568 (ovarian cancer, OV). In each dataset, the miRNA expression values were Z-normalized.

In the prospective serum validation phase, serum specimens were collected from 176 patients with GC and 173 healthy participants, matched by age and sex, who were prospectively recruited from March 2017 to August 2018 at Affiliated Hospital of Jiangsu University and Nantong Tumor Hospital, Jiangsu, China (**Table 1**).

© 2021 Izumi D et al. *JAMA Network Open.*

Prospective serum validation cohort

For prospective serum validation phase, patients who had a history of recurrent or other metastatic cancer were excluded from this study. Blood samples were taken when the patient was diagnosed with GC for the very first time without any treatment. Diagnosis was made by means of imaging techniques (computed tomography or magnetic resonance) and blood examination and verified by histopathological examination. All patients provided informed consent and met the inclusion criteria when enrolled in the study. CEA and CA19-9 levels were estimated in the serum specimens for patients with GC and healthy participants based on Cobase 601 (Roche Diagnostics) with Roche original reagents (ref. 11731629 and 11776193 respectively). Using qRT-PCR data for the 3-miRNA signature, the risk-scoring formula established in the previous cohort was applied and the performance of these circulating miRNA biomarkers was interrogated.

Sample collection and processing

Whole blood was collected in the blood collection tube without clot activator and without anticoagulants and left at room temperature (15-25°C) until complete clotting. The serum was transferred to a new tube after centrifuging for several times. After the serum was separated from the whole blood, it was stored at -80°C immediately for further processing.

Genome-wide miRNA data analysis

In the discovery phase, we first analyzed genome-wide miRNA sequencing data from TCGA (discovery cohort) to identify candidate miRNAs for the early detection of patients with GC. More specifically, level-3 miRNA expression data, including 436 tumors and 41 normal tissues, was downloaded from Firehose Broad GDAC portal (http://gdac.broadinstitute.org/, accessed on Nov 1, 2015). The miRNA expression levels, measured by reads per million miRNAs mapped (RPM), were

first log2-transformed. Differential miRNA expression analysis was performed between GC and adjacent normal tissues using the bioconductor package and "limma" package in R (*34*). To further evaluate the predictive power of each miRNA's expression level in distinguishing GC from normal tissue, AUC was calculated. Among 1046 targets, 104 were differentially expressed (BH-adjusted $P < 0.05$, absolute log2 fold change > 1 (*34*)) between GC and normal tissues. Among these, 9 miRNAs (miR-21, -196a-1, -146b, -196b, -135b, -181b, -181a, -93 and -335) were further selected based on the following criteria: BH-adjusted $P<1 \times 10^{-5}$, AUC > 0.9 and upregulated in GC. In addition, we also included two additional miRNAs, miR-196a-2 and 18a due to their high discriminative power (AUC=0.90 and 0.87, respectively), and because they were highly overexpressed in GC patients (log2 fold change >2, BH-adjusted $P < 1 \times 10^{-5}$). Due to the annotation differences between platforms, we combined expression data of miR-196a-1 and miR-196a-2 together as a single miRNA probe (miR-196a), which led to reduction of the 11-miRNA candidates to 10 miRNAs. 10 differentially expressed and discriminatory miRNAs between GC and normal tissues were selected as the initial signature candidates.

To confirm their diagnostic potential, the 10 miRNAs were validated in two additional independent datasets. GSE23739 included 40 GC and 40 non-cancerous tissue specimens with miRNA profiling data acquired using the Agilent-019118 Human miRNA Microarray platform (*35*). GSE33743 included 37 primary GC tissues and 4 normal gastric mucosa profiled for miRNA expression using the miRNAChip_human_V2 miRNA microarrays (National DNA-Microarray Facility) containing 1175 probes(*36*). The miRNA expression data was downloaded from GEO using Bioconductor package "GEOquery" in R, which was subsequently preprocessed using the methods described by Carvalho and colleagues (*35, 36*).

miRNA-mRNA regulatory network interaction and functional analysis

A miRNA-mRNA network was constructed to study the functional significance of the candidate

© 2021 Izumi D et al. *JAMA Network Open.*

miRNAs. Specifically, for each miRNA, its downstream target mRNAs was identified based on two key criteria: first, that each miRNA-mRNA interaction had been experimentally validated based on the miRTarBase database (V8) (*21*); second, that each downstream mRNA was differentially expressed between tumor and normal samples (|log2 fold change| > 2 & BH-adjusted $P < 0.05$) in the TCGA dataset. The functional analysis was performed based on hypergeometric tests using the "clusterProfiler" package (*37*), with C2 (curated gene sets) and C5 (GO and KEGG gene sets) collections retrieved from the MSigDB Database (v7.0) (*38*). *P* values were corrected for multiple hypothesis testing using the BH procedure, and a BH-adjusted $P < 0.0001$ was considered statistically significant.

Random Forest classification

To further evaluate the robustness of 10-miRNA signature across different datasets, a Random Forest classifier was trained using the expression levels of the 10 miRNAs by analyzing results from 41 GC and matched adjacent normal tissues in the discovery dataset. Z-normalization was performed for each miRNA separately in both validation datasets. Using the trained Random Forest classifier, predictive probabilities were calculated for identifying patients with GC, and the diagnostic performance of the combination of 10 miRNA candidates was assessed based on the AUC for both validation datasets.

Cost-effectiveness analysis

CE analysis was performed under the following clinical assumptions **(Supplementary Table 11)**: Non-invasive screening was assumed to be performed on a high-risk population, Chinese men between ages 50–75 years old. The compliance rate was estimated to be approximately 45%. The test-positive group were assumed to go on to have a confirmatory test using endoscopy and biopsy. The biopsy test is considered the gold standard, with 100% sensitivity and specificity. The test-negative group were assumed to have a 3-year follow-up, during which cancer patients would be detected during the follow-up period. For the non-screening group, 10% of high-risk population were estimated to receive an

© 2021 Izumi D et al. *JAMA Network Open.*

endoscopy test to evaluate the incidence of cancer. Due to the high sensitivity and specificity of the 3-miRNA signature, the rate of early-stage patients diagnosed was estimated to rise. Using the miRNA test in a large-scale screening was estimated to increase the detection rate of early-stage GC, which is calculated from the sensitivity and specificity of the 3-miRNA signature under the assumed compliance rate.

For the assumption of cancer treatment, early or advanced stages of GC (TNM Stage 1–3) were considered curable and would be cured after two years with a stage-specific recurrence rate. For cured patients, we estimated they would have additional medical expenditure (1000 CNY or 142.6 USD) every year before recurrence. Terminal stage GC (TNM Stage 4) was considered as untreatable, with only palliative care, and patients were assumed to die after one year. Considering the prognosis following cancer recurrence is poor, all relapsed patients were assumed to have Stage 4 status. Cost and incidence rate were either collected from the literature or estimated based on in-house clinical records**.**

RNA isolation and qRT-PCR

miRNA extraction from tissue specimens was performed using miRNeasy RNA isolation kits (Qiagen, Valencia, California, USA), whereas miRNA extraction from serum was performed using miRNeasy serum/plasma kits (Qiagen). TaqMan miRNA real-time qRT-PCR assays (Applied Biosystems, Foster City, California, USA) were used to detect and quantify miRNA expression. The expression levels of serum or tissue miRNAs were normalized against miR-16 and U6 expression levels, respectively, and results were calculated using the ΔCt method as previously described (*39*). To ensure consistent measurements throughout all assays, for each PCR amplification reaction, three independent RNA samples were loaded as internal controls to account for potential plate-to-plate variation, and the results from each plate were normalized against the internal normalization controls. All experiments were triplicated.

© 2021 Izumi D et al. *JAMA Network Open.*

Total RNA enriched in small RNAs was purified from serum samples using the miRNeasy Serum/Plasma Kit (Qiagen). C. elegans miR-39 miRNA mimic (miRNeasy Serum/Plasma Spike-In Control, Qiagen) was mixed thoroughly into all samples during the RNA isolation procedures for normalization of sample-to-sample variation. RNA extracted from serum samples was reverse-transcribed using a TaqMan MicroRNA Reverse Transcription Kit (Applied Biosystems) for subsequent qRT-PCR assays. qRT- PCR was performed using a TaqMan MicroRNA Assay kit and TaqMan Universal Master Mix II, no UNG (Applied Biosystems) through QuantStudioTM 7 Flex Real-Time PCR System (Applied Biosystems). The expression of miRNAs was normalized against the average expression level of miR-16 and 423-5p (Applied Biosystems) in all serum samples. Whenever we used kits from companies, we followed the instruction from the manufactures.

Logistic regression analysis with elastic net regularization

Using 115 patients with GC and 115 healthy participants enrolled in the public serum cohort (GSE106817), we developed a 3-circulating-miRNA signature using logistic regression with elastic net regularization. Using the established risk scoring formula, we calculated risk probabilities for samples in the prospective serum cohort to evaluate the diagnostic performance.

Statistical analysis

All statistical analyses were performed using Medcalc V.12.3.0 (Broekstraat 52, 9030; Mariakerke, Belgium), GraphPad Prism V5.0 (GraphPad Software, San Diego, California, USA), and R (3.3.3, R Development Core Team, https://cran.r-project.org/). Differential miRNA expression analysis was performed using the 'limma' package in R, and the resulting *P* values were adjusted using Benjamini-Hochberg's method. Wilcoxon's signed-rank test, the Mann-Whitney U test, and the Kruskal-Wallis test were used to analyze miRNA expression data obtained from qRT-PCR experiments, and results were expressed as mean ± standard error. Silhouette width was calculated using R package 'cluster'

© 2021 Izumi D et al. *JAMA Network Open.*

with Euclidean distance. Pearson correlation coefficient was calculated by R package ‘stats’ and graphically displayed by R package ‘corrplot’. The principal components analysis and other statistical analyses including logistic regression were also performed using the “stats” package in R. AUCs derived from ROC curves were calculated with CIs using the “pROC” package in R; ROC curves were compared using DeLong’s test in the pROC package. The 10-miRNA Random Forest classifier was trained using the “caret” package in R. Logistic regression with elastic net regularization was performed using the “glmnet” package through the caret package in R, with default configuration of optimization. ORs were calculated using the “vcd” package in R.

© 2021 Izumi D et al. *JAMA Network Open.*

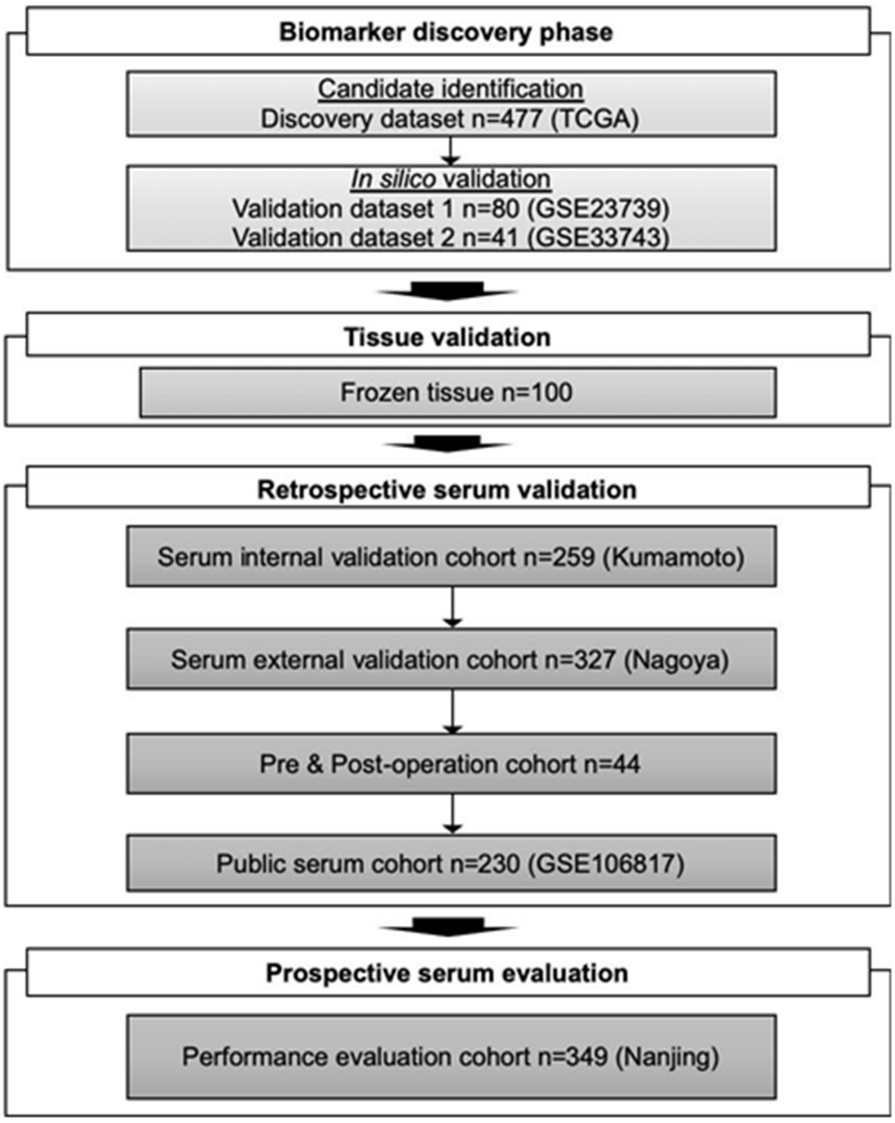

eFigure 1. Study design for the identification of a circulating miRNA expression signature for early detection of GC.

© 2021 Izumi D et al. *JAMA Network Open.*

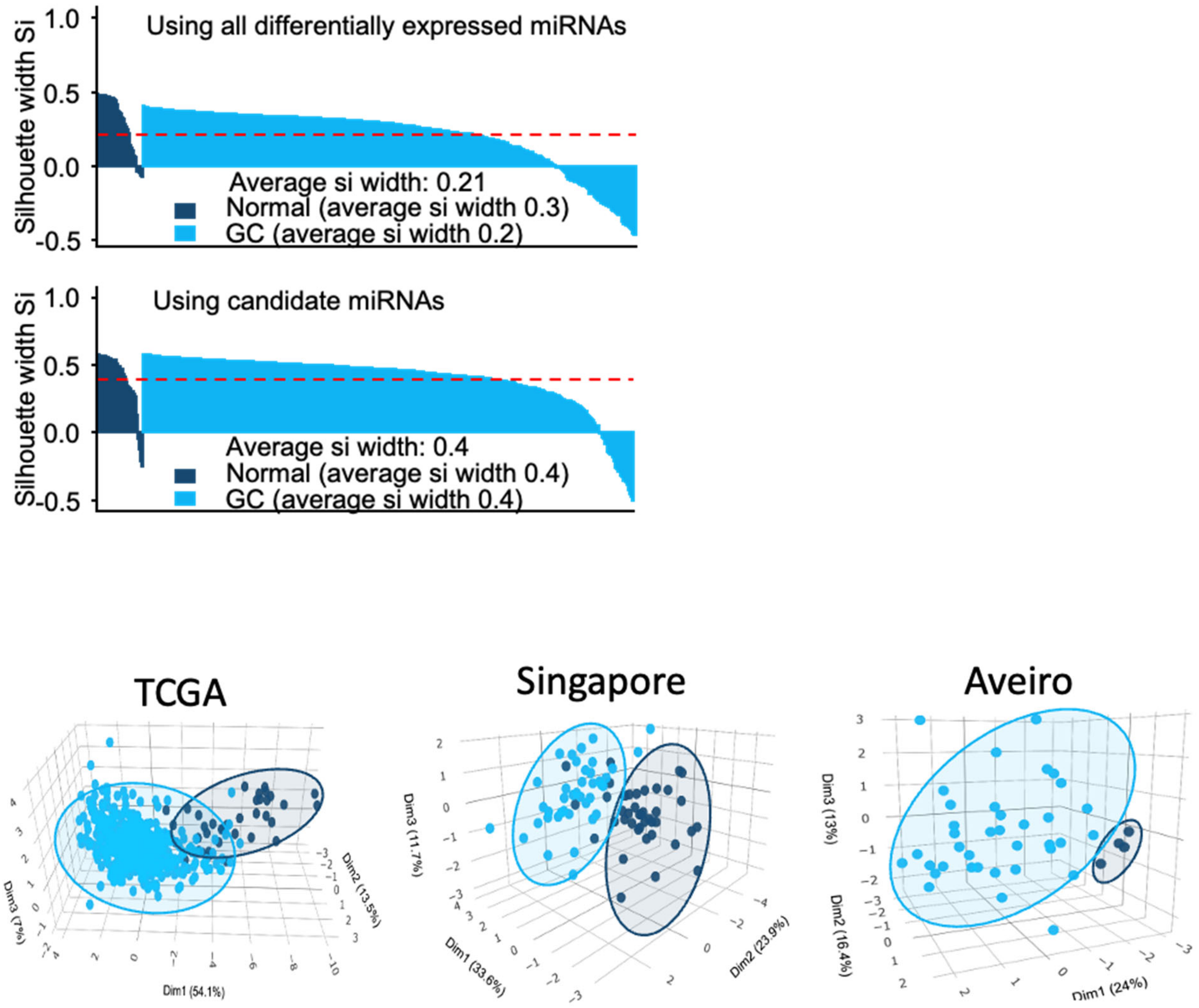

eFigure 2. Genome-wide discovery of miRNA candidates for GC diagnosis. (A) Using all differentially expressed miRNAs and 10 selected miRNAs, Silhouette analysis shows stability and consistency within GC and normal groups. Red dash line indicates the average silhouette width. (B) Principle component analysis shows 10 miRNAs can discriminate GC and normal samples.

© 2021 Izumi D et al. *JAMA Network Open.*

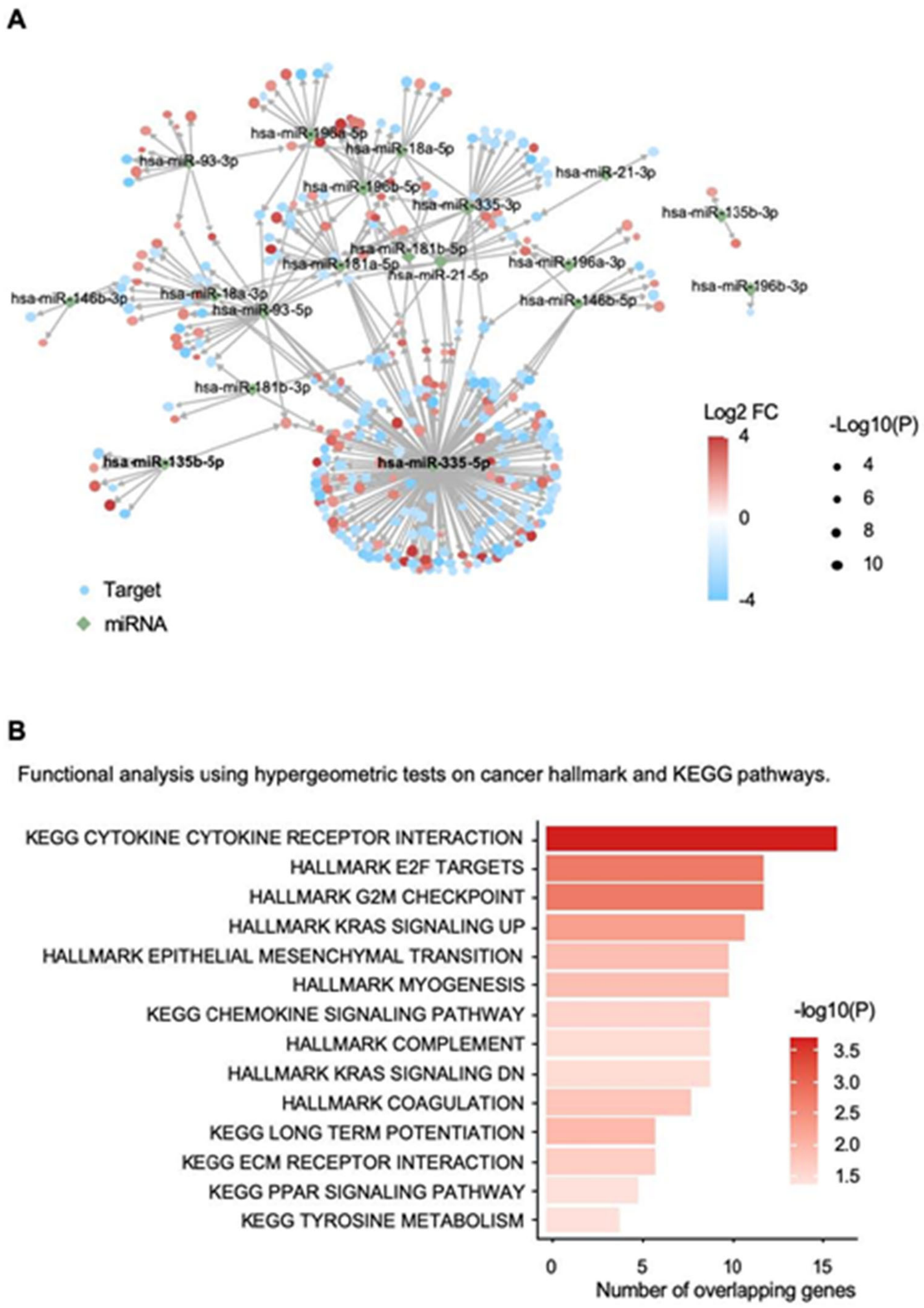

eFigure 3. miRNA regulatory network analysis and functional analysis of the miRNA target genes. (A) A regulatory network constructed using experimentally validated miRNA-mRNA interactions from miRTarBase (V8). Node size indicates the -log10 transformed BH-adjusted *P*, and color indicates the log2 fold change between GC and normal samples in the TCGA dataset. (B) Functional analysis using hypergeometric tests on cancer hallmark and KEGG pathways. The significantly enriched signaling pathways (BH-adjusted $P < 0.05$) are illustrated in the bar plot. Bar length indicates the number of overlapping genes and color indicates the *P* value.

© 2021 Izumi D et al. *JAMA Network Open.*

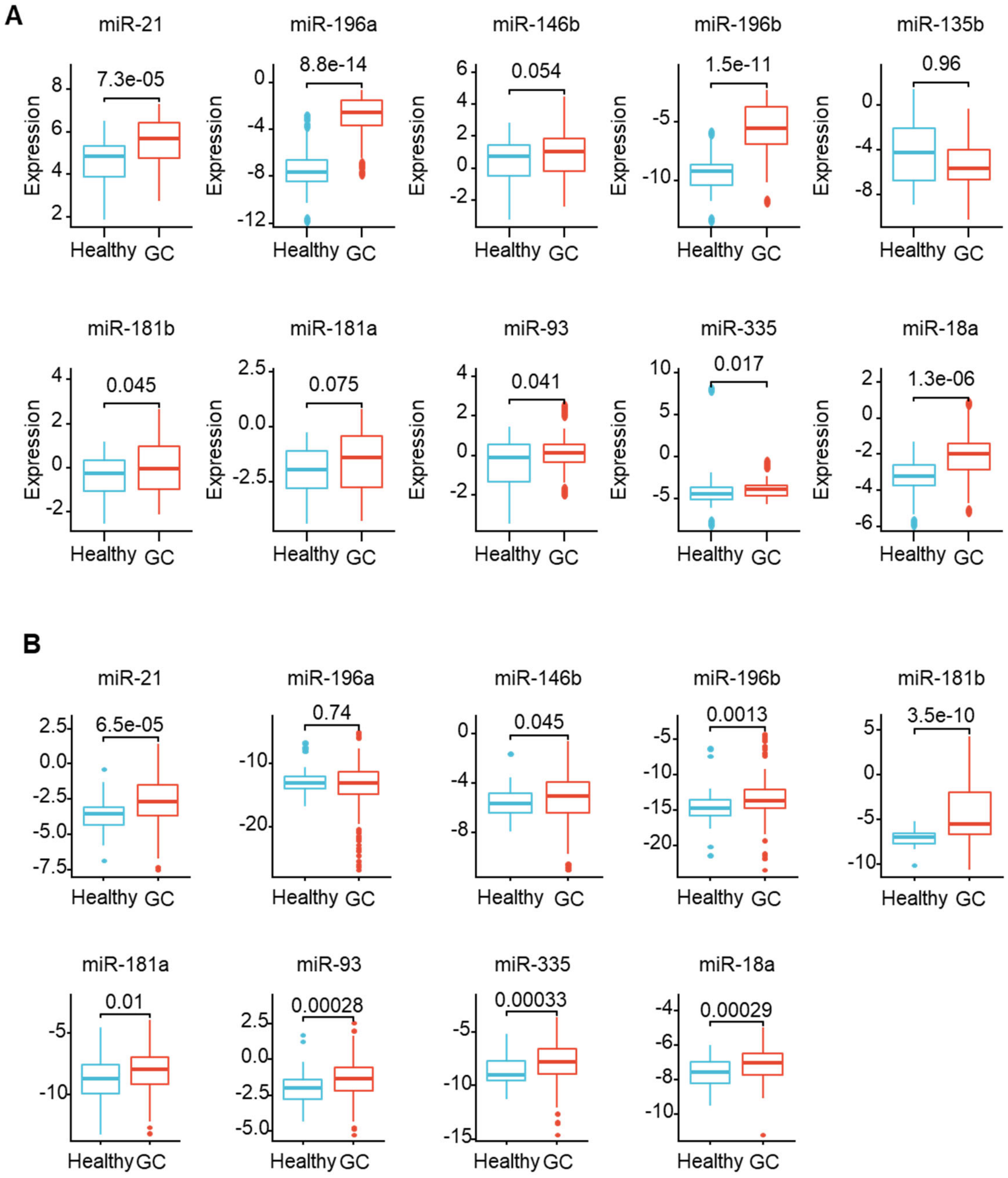

eFigure 4. Tissue validation and serum validation for the 10 miRNA candidates. (A) Boxplots with one-tailed Wilcoxon signed-rank tests comparing the expression levels between GC and adjacent healthy tissues. (B) Boxplots with Mann-Whitney U tests comparing the expression levels of the circulating miRNAs between patients with GC and healthy participants (Kumamoto cohort).

© 2021 Izumi D et al. *JAMA Network Open.*

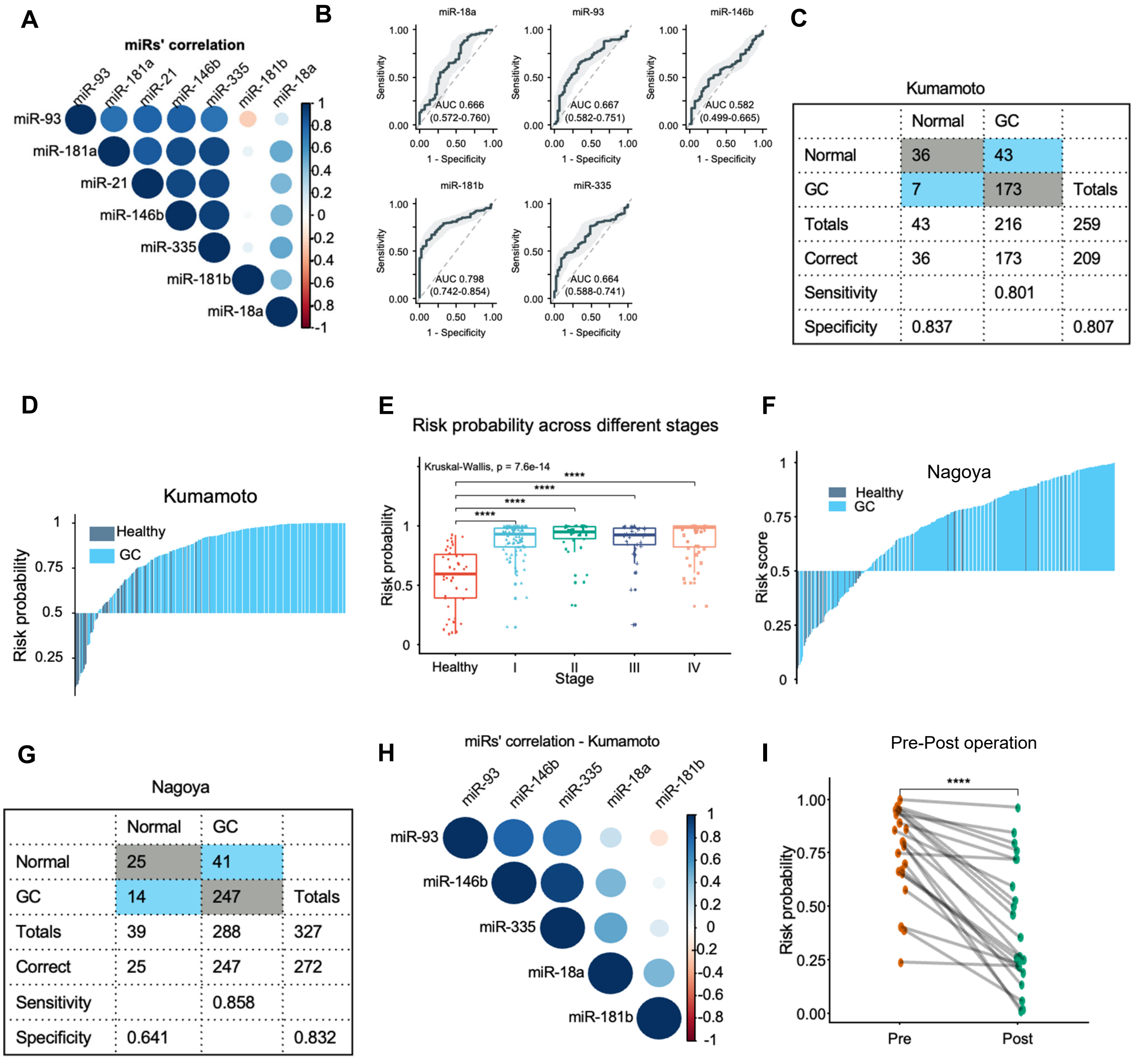

eFigure 5. Validation of the 5 circulating miRNAs in the Kumamoto cohort. (A) Correlation matrix of expression of seven miRNAs (miR-93, miR-181a, miR-21, miR-146b, miR-335, miR-181b and miR-18a) in the Kumamoto cohort. (B) ROC curves illustrating the diagnostic values of individual circulating miRNAs in differentiating patients with GC from healthy participants (Kumamoto cohort). (E) Confusion matrices built from diagnostic model prediction in the Kumamoto cohort. (D) Waterfall plot illustrating the detailed classification of patients with GC and participants using risk by risk probability in the Kumamoto cohort. (E) Boxplots comparing risk scores between patients with GC of different stages and healthy participants in the Kumamoto cohort. (F) Waterfall plot illustrating patients with GC and healthy participants ranked by risk probability in the serum external validation cohort (Nagoya). (G) Confusion matrices built from diagnostic model prediction in the Nagoya cohort. (H) Correlation matrix of expression of five miRNAs (miR-93, miR-146b, miR-335, miR-18a and miR-181b) in the Kumamoto cohort. (I) Paired dot plots comparing risk scores calculated for 22 pairs of pre- and post-operative sera form patients with GC. A significant drop in GC risk was observed after curative surgery (*P* < 0.0001, Wilcoxon signed-rank test), suggesting that expression of the circulating miRNAs was derived from GC tissues.

© 2021 Izumi D et al. *JAMA Network Open.*

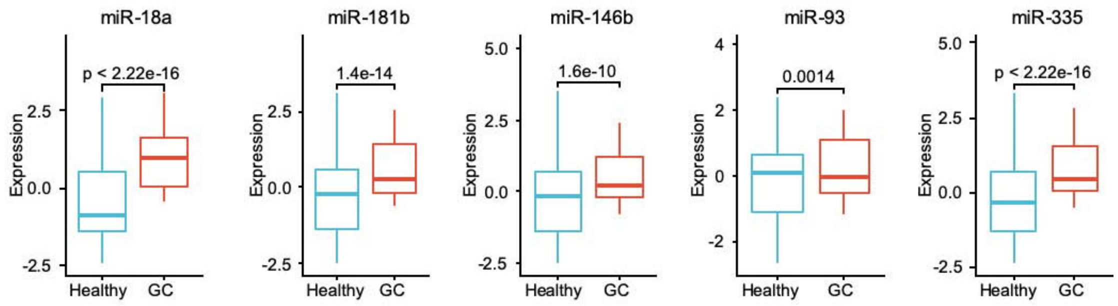

eFigure 6. Validation of the 5 circulating miRNAs in a public serum cohort (GSE106817). Boxplots with one-tailed Wilcoxon signed-rank tests comparing the expression levels of the circulating miRNAs between patients with GC and healthy participants.

© 2021 Izumi D et al. *JAMA Network Open.*

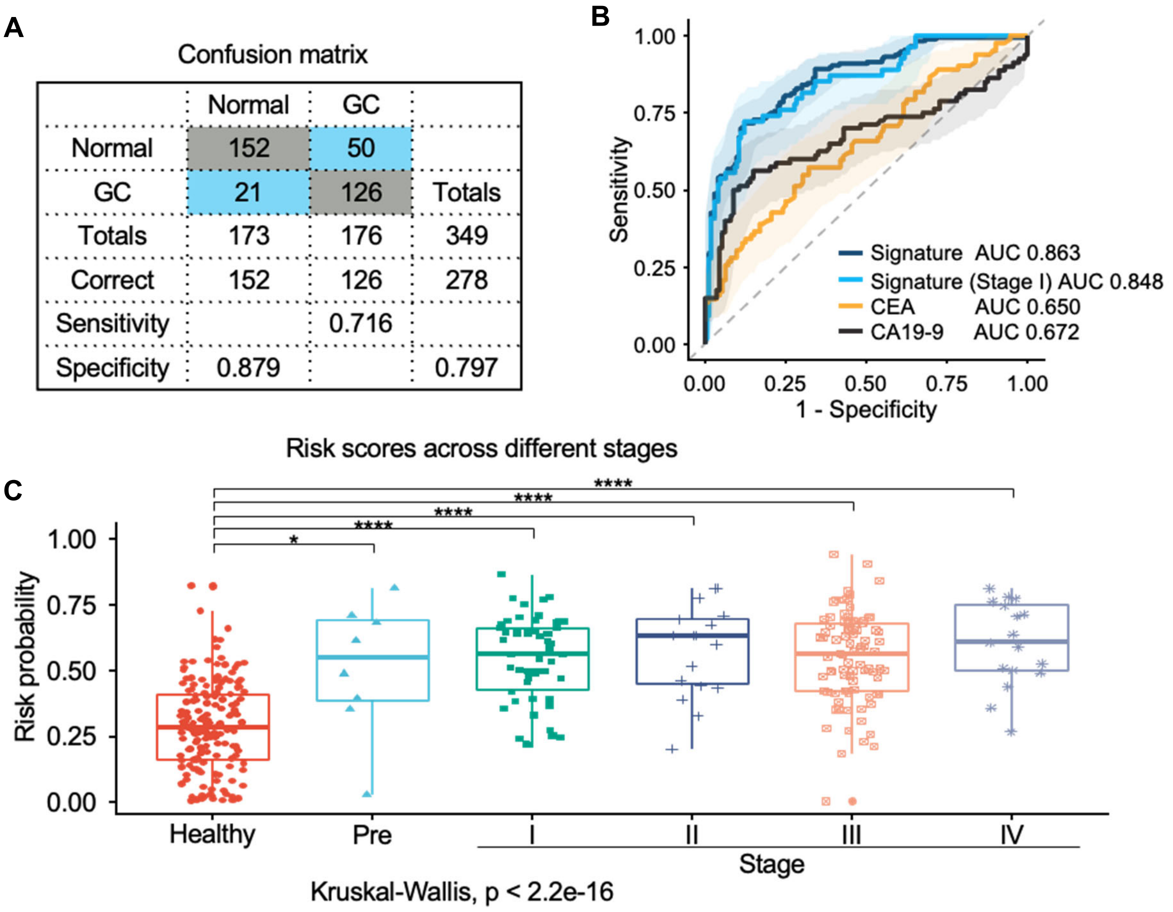

eFigure 7. Establishment of a 3-circulating-miRNA signature and evaluation in a prospective validation serum cohort. (A) Confusion matrices built from the diagnostic model prediction in the prospective validation cohort. (B) ROC curves demonstrating diagnostic performance of the 3-miRNA signature in all-stage GC samples, stage I GC samples, CEA and CA19-9 in a prospective validation cohort (Nanjing). ROC curves are shown with 95% CI (DeLong's test). (C) Boxplots comparing risk scores between patients with GC of different stages, precancerous lesion (pre) and healthy participants. All ROC curves are shown with 95% CI. The 95% CI of sensitivity and specificity (green line) for each miRNA is also shown at the best threshold (green point).

© 2021 Izumi D et al. *JAMA Network Open.*

eTable 1. Statistical summary of the initially identified 11 miRNAs from the discovery dataset

**miRNA**	**AUC**	**SEN**	**SPE**	**PPV**	**NPV**	**aveTumor**	**aveNormal**	**log2FC**	***P* value**	**FDR-adjusted *P* value**
hsa-mir-21	0.99	0.90	1.00	1.00	0.48	18.02	15.57	2.44	3.66E-25	3.19E-22
hsa-mir-196a-1	0.94	0.95	0.81	0.98	0.60	6.63	1.89	4.73	9.26E-21	8.05E-18
hsa-mir-146b	0.92	0.91	0.76	0.98	0.44	9.38	6.97	2.42	2.41E-19	2.09E-16
hsa-mir-196b	0.91	0.80	0.90	0.99	0.30	7.66	3.15	4.51	4.98E-18	4.31E-15
hsa-mir-135b	0.91	0.74	0.95	0.99	0.26	5.35	2.16	3.19	7.26E-18	6.29E-15
hsa-mir-181b-1	0.91	0.81	0.88	0.99	0.30	7.78	6.23	1.55	7.53E-18	6.51E-15
hsa-mir-181a-1	0.90	0.83	0.83	0.98	0.31	10.02	8.54	1.48	9.75E-18	8.41E-15
hsa-mir-93	0.90	0.73	1.00	1.00	0.26	12.28	10.50	1.78	1.68E-17	1.44E-14
hsa-mir-335	0.90	0.79	0.90	0.99	0.29	6.43	4.39	2.04	1.96E-17	1.68E-14
hsa-mir-196a-2	0.90	0.85	0.83	0.98	0.34	3.02	0.51	2.51	2.63E-17	2.25E-14
hsa-mir-18a	0.87	0.67	0.90	0.99	0.21	4.85	2.76	2.09	1.01E-14	8.56E-12

AUC, area under the ROC curve. SEN, sensitivity. SPE, specificit. PPV, positive predictive value. NPV, negative predictive value. ave, average. FC, fold change

© 2021 Izumi D et al. *JAMA Network Open.*

eTable 2. MiRNA-mRNA interactions in the regulatory network

miRNA	miRTarBase ID	Target Gene	log2FC	BH-adjusted *P* value
hsa-miR-21-5p	MIRT000171	NCAPG	2.38	3.79E-16
hsa-miR-181b-5p	MIRT000240	CDX2	3.11	2.78E-05
hsa-miR-181a-5p	MIRT000245	CDX2	3.11	2.78E-05
hsa-miR-21-5p	MIRT002416	BTG2	-2.01	4.03E-23
hsa-miR-196a-5p	MIRT002942	HOXC8	4.04	8.00E-14
hsa-miR-181b-5p	MIRT003035	GRIA2	-2.59	2.16E-09
hsa-miR-21-5p	MIRT003567	SERPINB5	2.02	8.38E-04
hsa-miR-135b-5p	MIRT003594	IBSP	4.24	1.00E-21
hsa-miR-18a-5p	MIRT004387	TNFSF11	2.76	5.71E-14
hsa-miR-196b-5p	MIRT004638	HOXC8	4.04	8.00E-14
hsa-miR-146b-5p	MIRT005371	KIT	-2.18	3.91E-15
hsa-miR-335-5p	MIRT006110	TFF2	-2.26	2.56E-03
hsa-miR-196a-5p	MIRT006803	HMGA2	2.93	3.84E-07
hsa-miR-335-5p	MIRT007304	BIRC5	2.24	3.78E-13
hsa-miR-335-5p	MIRT016709	NPY	-3.29	3.33E-12
hsa-miR-335-5p	MIRT016712	USH1C	2.08	5.20E-07
hsa-miR-335-5p	MIRT016727	DNASE1L3	-2.71	2.37E-16
hsa-miR-335-5p	MIRT016748	SYNPO2	-3.13	4.96E-14
hsa-miR-335-5p	MIRT016769	CCL15	2.52	3.58E-06
hsa-miR-335-5p	MIRT016828	LRP1B	-2.15	3.21E-09
hsa-miR-335-5p	MIRT016831	PRKN	-2.13	1.60E-20
hsa-miR-335-5p	MIRT016848	WNT7B	3.49	2.69E-07
hsa-miR-335-5p	MIRT016852	F10	-2.26	2.73E-12
hsa-miR-335-5p	MIRT016866	SPP1	3.09	1.75E-07
hsa-miR-335-5p	MIRT016876	MAPK4	-3.60	5.61E-14
hsa-miR-335-5p	MIRT016899	SORBS2	-2.00	1.55E-13
hsa-miR-335-5p	MIRT016901	NEFM	-2.58	9.17E-14
hsa-miR-335-5p	MIRT016917	HTR7	-2.21	8.12E-15
hsa-miR-335-5p	MIRT016925	TUSC5	-3.75	4.86E-18
hsa-miR-335-5p	MIRT016935	SALL4	4.04	2.35E-12
hsa-miR-335-5p	MIRT016944	CXCL9	2.49	2.08E-06
hsa-miR-335-5p	MIRT016951	VIP	-3.49	1.63E-12
hsa-miR-335-5p	MIRT016968	NECAB1	-2.21	1.17E-12
hsa-miR-335-5p	MIRT016993	GRB7	2.35	6.71E-08
hsa-miR-335-5p	MIRT017035	CYP19A1	2.27	3.06E-09
hsa-miR-335-5p	MIRT017058	ASB11	-2.38	9.34E-11
hsa-miR-335-5p	MIRT017063	SLC2A12	-2.16	1.17E-12
hsa-miR-335-5p	MIRT017068	HSPB3	-3.14	6.55E-13
hsa-miR-335-5p	MIRT017072	HOXA11	4.96	6.38E-11
hsa-miR-335-5p	MIRT017114	FLG2	-2.20	1.23E-09
hsa-miR-335-5p	MIRT017120	FOXA2	2.13	6.15E-05
hsa-miR-335-5p	MIRT017127	C6orf223	3.11	3.65E-07

© 2021 Izumi D et al. *JAMA Network Open.*

hsa-miR-335-5p	MIRT017144	HOXC6	2.54	5.00E-09
hsa-miR-335-5p	MIRT017145	MMRN1	-2.86	1.31E-18
hsa-miR-335-5p	MIRT017148	WIF1	-3.90	1.76E-15
hsa-miR-335-5p	MIRT017159	SFRP1	-4.23	2.09E-25
hsa-miR-335-5p	MIRT017193	CPNE6	-2.84	9.58E-13
hsa-miR-335-5p	MIRT017225	PYGM	-3.05	7.10E-24
hsa-miR-335-5p	MIRT017231	SLC27A6	-2.62	2.81E-11
hsa-miR-335-5p	MIRT017241	IYD	2.57	2.42E-05
hsa-miR-335-5p	MIRT017263	ILDR1	2.71	4.38E-15
hsa-miR-335-5p	MIRT017272	SERPINB5	2.02	8.38E-04
hsa-miR-335-5p	MIRT017286	PLA2G7	2.82	6.83E-15
hsa-miR-335-5p	MIRT017304	PGM5	-3.67	9.10E-23
hsa-miR-335-5p	MIRT017308	IGSF10	-2.16	2.67E-10
hsa-miR-335-5p	MIRT017316	GRIN2D	3.39	1.17E-12
hsa-miR-335-5p	MIRT017317	LONRF2	-2.61	1.05E-10
hsa-miR-335-5p	MIRT017342	COL10A1	5.83	1.17E-12
hsa-miR-335-5p	MIRT017349	DMBX1	3.86	8.42E-12
hsa-miR-335-5p	MIRT017350	PPP1R3C	-2.95	1.49E-19
hsa-miR-335-5p	MIRT017371	PLIN4	-3.82	1.19E-19
hsa-miR-335-5p	MIRT017377	TMEFF2	-2.55	2.33E-11
hsa-miR-335-5p	MIRT017386	SCG3	-2.67	9.76E-10
hsa-miR-335-5p	MIRT017395	CLDN4	2.52	1.32E-10
hsa-miR-335-5p	MIRT017398	R3HDML	2.55	3.71E-12
hsa-miR-335-5p	MIRT017399	ABCA8	-2.99	4.54E-16
hsa-miR-335-5p	MIRT017405	SULT2A1	-3.00	1.45E-07
hsa-miR-335-5p	MIRT017414	COL11A1	4.94	1.34E-09
hsa-miR-335-5p	MIRT017431	CELSR3	2.20	2.30E-10
hsa-miR-335-5p	MIRT017434	XKR4	-3.14	6.35E-18
hsa-miR-335-5p	MIRT017438	UBD	2.83	8.46E-08
hsa-miR-335-5p	MIRT017445	SNCG	-2.21	1.89E-14
hsa-miR-335-5p	MIRT017448	MUC3A	2.41	1.15E-04
hsa-miR-335-5p	MIRT017451	CDH3	2.77	2.64E-10
hsa-miR-335-5p	MIRT017452	GPIHBP1	-2.06	1.17E-12
hsa-miR-335-5p	MIRT017453	HTRA4	2.92	7.96E-16
hsa-miR-335-5p	MIRT017464	LY6H	-2.39	1.60E-13
hsa-miR-335-5p	MIRT017510	CLDN1	3.08	1.17E-12
hsa-miR-335-5p	MIRT017511	ENTPD3	-2.51	1.17E-12
hsa-miR-335-5p	MIRT017526	SMYD1	-3.17	1.74E-06
hsa-miR-335-5p	MIRT017560	CHRM2	-3.47	8.25E-10
hsa-miR-335-5p	MIRT017565	RSPO1	-2.41	2.68E-10
hsa-miR-335-5p	MIRT017577	CST4	3.72	4.03E-22
hsa-miR-335-5p	MIRT017599	RPS6KA6	-2.96	3.42E-17
hsa-miR-335-5p	MIRT017610	4	-2.82	2.13E-09
hsa-miR-335-5p	MIRT017613	TERT	2.36	9.14E-07
hsa-miR-335-5p	MIRT017615	SLC17A7	-2.02	4.33E-13

© 2021 Izumi D et al. *JAMA Network Open.*

hsa-miR-335-5p	MIRT017622	FOXD3	-2.91	1.30E-12
hsa-miR-335-5p	MIRT017688	CCL14	-2.09	4.33E-12
hsa-miR-335-5p	MIRT017697	FNDC1	3.08	7.87E-08
hsa-miR-335-5p	MIRT017702	C6orf58	-3.03	1.92E-07
hsa-miR-335-5p	MIRT017703	LRAT	-2.80	1.54E-10
hsa-miR-335-5p	MIRT017705	LAIR2	2.01	6.12E-06
hsa-miR-335-5p	MIRT017706	GUCA2B	-2.87	1.39E-08
hsa-miR-335-5p	MIRT017708	PRKCG	2.55	7.16E-06
hsa-miR-335-5p	MIRT017731	CLEC3B	-3.69	3.17E-49
hsa-miR-335-5p	MIRT017735	KIT	-2.18	3.91E-15
hsa-miR-335-5p	MIRT017751	BVES-AS1	-2.70	2.90E-13
hsa-miR-335-5p	MIRT017769	TSLP	-2.45	2.13E-17
hsa-miR-335-5p	MIRT017782	MISP	2.73	5.58E-12
hsa-miR-335-5p	MIRT017841	TACR2	-2.96	4.57E-11
hsa-miR-335-5p	MIRT017846	TREH	-2.17	2.45E-11
hsa-miR-335-5p	MIRT017858	SOX9	2.59	3.03E-19
hsa-miR-335-5p	MIRT017862	LIPG	2.10	1.55E-09
hsa-miR-335-5p	MIRT017878	CLDN7	2.21	3.47E-12
hsa-miR-335-5p	MIRT017902	ENDOU	-2.79	5.65E-14
hsa-miR-335-5p	MIRT017921	SORCS3	-2.78	3.25E-12
hsa-miR-335-5p	MIRT017927	PNLIPRP3	-2.61	5.36E-08
hsa-miR-335-5p	MIRT017934	PEBP4	-3.66	7.13E-27
hsa-miR-335-5p	MIRT017935	GSG1L	-2.27	4.09E-14
hsa-miR-335-5p	MIRT017936	WDR17	-2.26	2.37E-10
hsa-miR-335-5p	MIRT017953	PABPC1L	2.21	1.51E-12
hsa-miR-335-5p	MIRT017984	XIRP1	2.57	9.35E-08
hsa-miR-335-5p	MIRT017988	SMOC2	-2.04	1.80E-09
hsa-miR-335-5p	MIRT018001	CTHRC1	3.10	8.77E-15
hsa-miR-335-5p	MIRT018039	SOSTDC1	-4.41	1.61E-23
hsa-miR-335-5p	MIRT018041	TRIM50	-3.10	1.90E-12
hsa-miR-335-5p	MIRT018048	LGALS9C	-2.70	9.96E-12
hsa-miR-335-5p	MIRT018057	LIF	2.14	2.44E-12
hsa-miR-335-5p	MIRT018066	MAL2	2.19	1.43E-12
hsa-miR-335-5p	MIRT018068	GSN	-2.06	1.62E-30
hsa-miR-335-5p	MIRT018085	ATP6V0D2	2.39	1.89E-08
hsa-miR-335-5p	MIRT018126	LMOD1	-3.28	1.48E-18
hsa-miR-335-5p	MIRT018192	NOVA1	-2.36	1.17E-12
hsa-miR-335-5p	MIRT018205	ANK2	-2.12	2.99E-11
hsa-miR-335-5p	MIRT018209	CRNN	-2.84	4.39E-04
hsa-miR-335-5p	MIRT018220	CCDC69	-2.36	1.03E-22
hsa-miR-335-5p	MIRT018249	ADAMTS12	2.90	5.65E-14
hsa-miR-335-5p	MIRT018277	CST1	6.74	1.17E-12
hsa-miR-335-5p	MIRT018278	CXCL3	2.13	7.27E-06
hsa-miR-335-5p	MIRT018281	PDZD4	-2.78	2.10E-17
hsa-miR-335-5p	MIRT018310	PPP1R1A	-4.21	1.12E-23

hsa-miR-335-5p	MIRT018332	BCHE	-3.24	3.41E-17
hsa-miR-335-5p	MIRT018333	GIP	-3.53	1.46E-12
hsa-miR-335-5p	MIRT018336	HS6ST3	-3.15	2.90E-13
hsa-miR-335-5p	MIRT018338	APOC1	3.10	1.09E-12
hsa-miR-335-5p	MIRT018340	HAVCR1	2.30	4.13E-04
hsa-miR-335-5p	MIRT018353	NPY1R	-2.13	2.96E-07
hsa-miR-335-5p	MIRT018375	PKD2L1	2.81	1.21E-16
hsa-miR-335-5p	MIRT018397	BEX2	-2.51	2.93E-08
hsa-miR-335-5p	MIRT018430	ASCL2	2.29	1.06E-04
hsa-miR-335-5p	MIRT018431	SNAP91	-2.32	3.25E-07
hsa-miR-335-5p	MIRT018443	RASGEF1C	-2.26	3.47E-11
hsa-miR-335-5p	MIRT018447	ABCC8	-2.30	5.58E-09
hsa-miR-335-5p	MIRT018455	FOXD2	2.32	2.44E-13
hsa-miR-335-5p	MIRT018459	BAAT	3.94	2.78E-08
hsa-miR-335-5p	MIRT018464	MLN	-2.23	3.70E-05
hsa-miR-335-5p	MIRT018484	CHST9	-3.07	2.25E-09
hsa-miR-335-5p	MIRT018515	CDH19	-3.91	7.16E-18
hsa-miR-335-5p	MIRT018520	DEFB4A	-2.47	1.84E-05
hsa-miR-335-5p	MIRT018522	GJB4	2.46	2.89E-05
hsa-miR-335-5p	MIRT018542	ZBTB7C	-2.24	2.28E-09
hsa-miR-335-5p	MIRT018544	FGF10	-2.03	4.82E-07
hsa-miR-335-5p	MIRT018574	GATA5	-2.03	1.55E-03
hsa-miR-335-5p	MIRT018587	CHL1	-2.14	4.19E-12
hsa-miR-335-5p	MIRT018593	SCARA5	-4.54	3.45E-35
hsa-miR-335-5p	MIRT018598	NT5C1A	-3.11	8.57E-23
hsa-miR-335-5p	MIRT018642	GCNT4	-2.93	3.73E-22
hsa-miR-335-5p	MIRT018670	CDX2	3.11	2.78E-05
hsa-miR-335-5p	MIRT018703	GDF15	3.13	3.48E-13
hsa-miR-335-5p	MIRT018735	CGNL1	-2.12	3.40E-13
hsa-miR-335-5p	MIRT018740	ADH7	-6.35	4.69E-32
hsa-miR-335-5p	MIRT018757	TPO	-2.09	8.20E-08
hsa-miR-335-5p	MIRT018762	LGALS9B	-2.78	1.38E-11
hsa-miR-335-5p	MIRT018772	COL4A5	-2.42	2.89E-13
hsa-miR-335-5p	MIRT018779	CNTD2	2.23	8.93E-06
hsa-miR-335-5p	MIRT018781	FGL2	-2.13	3.18E-13
hsa-miR-335-5p	MIRT018787	PEG3	-2.12	1.09E-11
hsa-miR-335-5p	MIRT018793	ADGRB3	-2.61	1.21E-12
hsa-miR-335-5p	MIRT018814	HPDL	2.06	1.01E-07
hsa-miR-335-5p	MIRT018815	ASB2	-2.66	1.09E-15
hsa-miR-335-5p	MIRT018818	CST2	3.06	3.37E-08
hsa-miR-335-5p	MIRT018820	ADGRG4	-3.48	4.54E-17
hsa-miR-335-5p	MIRT018828	AQP10	-3.63	7.75E-16
hsa-miR-335-5p	MIRT018837	EMILIN3	-2.83	1.12E-25
hsa-miR-335-5p	MIRT018862	SLC6A4	-2.76	2.22E-17
hsa-miR-335-5p	MIRT018894	ENHO	-2.19	9.38E-10

© 2021 Izumi D et al. *JAMA Network Open.*

hsa-miR-335-5p	MIRT018900	DCSTAMP	3.05	6.17E-14
hsa-miR-335-5p	MIRT018910	PDK4	-3.08	2.84E-21
hsa-miR-335-5p	MIRT018916	CPE	-2.15	7.07E-15
hsa-miR-335-5p	MIRT018933	CD36	-2.13	2.05E-16
hsa-miR-335-5p	MIRT018952	CALML5	-2.09	3.14E-04
hsa-miR-335-5p	MIRT018965	RSPO2	-3.59	1.90E-19
hsa-miR-335-5p	MIRT018980	PCSK9	2.11	8.38E-05
hsa-miR-335-5p	MIRT018981	VEGFD	-4.15	9.22E-41
hsa-miR-335-5p	MIRT018996	GNGT1	2.28	6.00E-04
hsa-miR-335-5p	MIRT019004	FHL1	-3.31	1.26E-25
hsa-miR-335-5p	MIRT019012	CPO	-2.31	2.55E-13
hsa-miR-335-5p	MIRT019032	CXCL8	2.72	1.46E-06
hsa-miR-335-5p	MIRT019040	FABP4	-3.47	1.13E-20
hsa-miR-335-5p	MIRT019060	HMGCLL1	-2.78	3.35E-16
hsa-miR-335-5p	MIRT019062	SFTPD	-2.14	2.07E-08
hsa-miR-335-5p	MIRT019069	ANKS4B	2.11	3.66E-05
hsa-miR-335-5p	MIRT019084	GPER1	-2.62	9.25E-18
hsa-miR-335-5p	MIRT019097	DIRAS1	-2.74	1.17E-14
hsa-miR-335-5p	MIRT019104	SPINK7	-3.88	3.56E-13
hsa-miR-335-5p	MIRT019110	ATP1B2	-2.02	4.21E-14
hsa-miR-335-5p	MIRT019111	SLC2A4	-3.08	4.25E-21
hsa-miR-335-5p	MIRT019119	FAM189A1	-2.56	2.36E-08
hsa-miR-335-5p	MIRT019120	RERGL	-2.90	5.10E-15
hsa-miR-335-5p	MIRT019174	TMEM132C	-3.85	7.31E-22
hsa-miR-335-5p	MIRT019185	GDF10	-2.73	1.48E-10
hsa-miR-335-5p	MIRT019192	MSLN	2.87	1.52E-04
hsa-miR-335-5p	MIRT019202	PKHD1L1	-2.68	2.19E-17
hsa-miR-335-5p	MIRT019216	ASTN1	-2.48	4.40E-08
hsa-miR-335-5p	MIRT019223	HTR1D	2.08	8.29E-04
hsa-miR-335-5p	MIRT019225	MYOT	-2.80	8.21E-19
hsa-miR-181a-5p	MIRT025043	FXYD6	-2.16	1.70E-14
hsa-miR-181a-5p	MIRT025054	PHOX2A	-2.66	2.99E-10
hsa-miR-181a-5p	MIRT025057	GSTM2	-2.06	7.77E-24
hsa-miR-181a-5p	MIRT025060	PTPRZ1	-2.65	5.37E-10
hsa-miR-181a-5p	MIRT025069	NMRK2	-2.32	1.75E-10
hsa-miR-181a-5p	MIRT025070	WNT2	4.09	5.04E-13
hsa-miR-181a-5p	MIRT025134	OFCC1	2.14	9.78E-12
hsa-miR-181a-5p	MIRT025148	CHL1	-2.14	4.19E-12
hsa-miR-181a-5p	MIRT025163	PCLAF	2.23	3.45E-15
hsa-miR-196a-5p	MIRT026059	IGF2BP3	3.23	2.17E-07
hsa-miR-196a-5p	MIRT026082	PRUNE2	-2.51	6.39E-10
hsa-miR-196a-5p	MIRT026092	EPHA7	-2.82	3.83E-10
hsa-miR-196a-5p	MIRT026098	IGF2BP1	3.25	1.36E-04
hsa-miR-93-5p	MIRT027944	TMEM100	-3.70	6.42E-26
hsa-miR-93-5p	MIRT027950	ZIC5	3.10	1.60E-04

© 2021 Izumi D et al. *JAMA Network Open.*

hsa-miR-93-5p	MIRT027969	FAM129A	-2.16	2.35E-14
hsa-miR-93-5p	MIRT028167	PRUNE2	-2.51	6.39E-10
hsa-miR-93-5p	MIRT028172	EPHA7	-2.82	3.83E-10
hsa-miR-21-5p	MIRT030694	SOX2	-2.30	1.88E-05
hsa-miR-21-5p	MIRT030776	LONRF2	-2.61	1.05E-10
hsa-miR-21-5p	MIRT030815	KNL1	2.19	5.78E-16
hsa-miR-21-5p	MIRT030819	FAXDC2	-2.26	5.40E-20
hsa-miR-21-5p	MIRT030871	MMP9	2.32	2.89E-08
hsa-miR-21-5p	MIRT030888	DMD	-2.05	2.08E-12
hsa-miR-21-5p	MIRT030914	TOP2A	2.79	3.08E-16
hsa-miR-21-5p	MIRT030986	HOXA9	2.85	2.84E-07
hsa-miR-21-5p	MIRT031012	OLR1	2.89	5.14E-12
hsa-miR-21-5p	MIRT031067	TNFRSF11B	2.07	1.16E-05
hsa-miR-18a-5p	MIRT031333	HOXA9	2.85	2.84E-07
hsa-miR-93-3p	MIRT038720	HASPIN	2.26	1.26E-16
hsa-miR-93-3p	MIRT038725	CSAG1	2.94	6.76E-04
hsa-miR-93-3p	MIRT038774	CENPF	2.76	2.92E-19
hsa-miR-93-3p	MIRT038799	HMMR	2.57	2.53E-18
hsa-miR-93-3p	MIRT038870	CDC20	2.20	1.17E-12
hsa-miR-18a-3p	MIRT040758	GINS1	2.11	2.01E-16
hsa-miR-18a-3p	MIRT040765	C6orf222	2.75	5.26E-07
hsa-miR-18a-3p	MIRT040818	CACNA2D3	-2.03	1.02E-13
hsa-miR-18a-3p	MIRT040857	CDCA2	2.69	3.41E-17
hsa-miR-18a-3p	MIRT040875	PTGS1	-2.10	8.56E-22
hsa-miR-18a-3p	MIRT040934	ASF1B	2.07	3.05E-14
hsa-miR-18a-3p	MIRT040971	CLU	-2.04	3.41E-10
hsa-miR-196b-5p	MIRT042665	SLC6A15	-2.53	7.84E-08
hsa-miR-196b-5p	MIRT042672	HTR1D	2.08	8.29E-04
hsa-miR-196b-5p	MIRT042674	CKB	-2.89	1.99E-21
hsa-miR-196b-5p	MIRT042691	BUB1	2.67	4.33E-20
hsa-miR-196a-5p	MIRT048156	NRXN1	-3.79	9.40E-22
hsa-miR-196a-5p	MIRT048176	REEP2	-2.08	2.54E-11
hsa-miR-196a-5p	MIRT048197	UBE2C	2.82	1.78E-15
hsa-miR-196a-5p	MIRT048215	CKAP2L	2.25	7.43E-18
hsa-miR-196a-5p	MIRT048234	KIF18B	3.06	1.88E-21
hsa-miR-196a-5p	MIRT048236	SH3GL3	-2.21	2.39E-07
hsa-miR-196a-5p	MIRT048279	BUB1	2.67	4.33E-20
hsa-miR-93-5p	MIRT048739	ZIC2	2.89	4.99E-04
hsa-miR-93-5p	MIRT048806	BIRC5	2.24	3.78E-13
hsa-miR-18a-5p	MIRT050708	CDC20	2.20	1.17E-12
hsa-miR-18a-5p	MIRT050725	PKMYT1	2.62	1.86E-16
hsa-miR-18a-5p	MIRT050732	CDCA5	2.33	7.22E-16
hsa-miR-181a-5p	MIRT052993	PGR	-2.11	1.37E-12
hsa-miR-93-5p	MIRT053085	SLC2A4	-3.08	4.25E-21
hsa-miR-21-5p	MIRT053173	HPGD	-2.95	1.79E-19

© 2021 Izumi D et al. *JAMA Network Open.*

hsa-miR-196b-5p	MIRT053330	HOXA9	2.85	2.84E-07
hsa-miR-146b-5p	MIRT053567	UHRF1	2.30	1.58E-16
hsa-miR-196b-5p	MIRT053586	HOXA10	4.24	2.35E-09
hsa-miR-181a-5p	MIRT053664	TNFRSF11B	2.07	1.16E-05
hsa-miR-181a-5p	MIRT053680	BMP3	-2.98	4.96E-08
hsa-miR-181a-5p	MIRT053690	ACAN	3.62	2.65E-18
hsa-miR-196b-5p	MIRT066479	HMGA2	2.93	3.84E-07
hsa-miR-93-5p	MIRT084345	RRM2	2.29	2.20E-14
hsa-miR-181b-3p	MIRT118893	LONRF2	-2.61	1.05E-10
hsa-miR-181a-5p	MIRT138427	KIF2C	2.05	1.34E-14
hsa-miR-181b-5p	MIRT138428	KIF2C	2.05	1.34E-14
hsa-miR-18a-5p	MIRT273157	RAD51AP1	2.11	8.77E-18
hsa-miR-18a-5p	MIRT282668	SYNM	-3.39	2.93E-15
hsa-miR-135b-5p	MIRT289438	BIRC5	2.24	3.78E-13
hsa-miR-146b-5p	MIRT437621	S100A12	-2.45	4.90E-10
hsa-miR-93-5p	MIRT437750	SLC16A9	-2.24	4.05E-09
hsa-miR-181a-5p	MIRT437924	TERT	2.36	9.14E-07
hsa-miR-181a-5p	MIRT437969	IFNG	2.00	1.96E-05
hsa-miR-93-5p	MIRT438087	CXCL8	2.72	1.46E-06
hsa-miR-21-5p	MIRT438112	CXCL10	2.14	9.57E-06
hsa-miR-21-5p	MIRT438224	CLU	-2.04	3.41E-10
hsa-miR-181a-5p	MIRT438437	WIF1	-3.90	1.76E-15
hsa-miR-18a-5p	MIRT442747	PTCHD1	-3.31	3.35E-15
hsa-miR-196b-3p	MIRT443008	KBTBD12	-2.09	8.79E-06
hsa-miR-146b-5p	MIRT448042	SFRP1	-4.23	2.09E-25
hsa-miR-135b-5p	MIRT448598	PCP4L1	-3.59	1.94E-15
hsa-miR-335-3p	MIRT449054	MGAT4C	-2.06	2.73E-07
hsa-miR-146b-5p	MIRT449334	ACTBL2	2.34	1.62E-07
hsa-miR-135b-5p	MIRT449577	APOA1	-2.69	2.71E-05
hsa-miR-93-5p	MIRT450918	CADM2	-3.87	5.33E-21
hsa-miR-18a-3p	MIRT467476	SMYD1	-3.17	1.74E-06
hsa-miR-93-5p	MIRT494434	BTG2	-2.01	4.03E-23
hsa-miR-93-5p	MIRT506852	KIF23	2.43	2.05E-20
hsa-miR-93-5p	MIRT506883	PCLAF	2.23	3.45E-15
hsa-miR-196a-5p	MIRT507497	E2F7	2.67	2.28E-16
hsa-miR-196b-5p	MIRT507498	E2F7	2.67	2.28E-16
hsa-miR-181b-3p	MIRT513264	SCUBE1	-2.44	3.81E-16
hsa-miR-93-5p	MIRT513708	RBM20	-2.31	1.25E-12
hsa-miR-93-5p	MIRT517183	SLC28A1	-2.41	1.36E-09
hsa-miR-93-5p	MIRT518695	KCNMB1	-2.79	6.47E-18
hsa-miR-146b-3p	MIRT526287	KY	-2.08	1.06E-11
hsa-miR-18a-3p	MIRT526291	KY	-2.08	1.06E-11
hsa-miR-93-3p	MIRT528115	FOXH1	2.68	4.51E-08
hsa-miR-181b-3p	MIRT529502	IYD	2.57	2.42E-05
hsa-miR-135b-3p	MIRT531366	SPC25	2.03	6.73E-15

© 2021 Izumi D et al. *JAMA Network Open.*

hsa-miR-181a-5p	MIRT535284	PHOX2B	-2.72	4.83E-10
hsa-miR-181b-5p	MIRT535285	PHOX2B	-2.72	4.83E-10
hsa-miR-335-5p	MIRT536189	MAOB	-2.47	4.00E-14
hsa-miR-196a-3p	MIRT540504	CXCL10	2.14	9.57E-06
hsa-miR-196b-5p	MIRT547839	IGF2BP3	3.23	2.17E-07
hsa-miR-196a-5p	MIRT547902	HOXA9	2.85	2.84E-07
hsa-miR-135b-5p	MIRT549355	ARC	-3.13	4.14E-15
hsa-miR-196b-5p	MIRT555194	PRUNE2	-2.51	6.39E-10
hsa-miR-181b-5p	MIRT557275	HMGA2	2.93	3.84E-07
hsa-miR-181a-5p	MIRT557276	HMGA2	2.93	3.84E-07
hsa-miR-335-3p	MIRT562743	AOC3	-2.15	1.10E-12
hsa-miR-93-5p	MIRT567057	KCNB1	-3.66	1.81E-18
hsa-miR-335-3p	MIRT569802	XKR4	-3.14	6.35E-18
hsa-miR-146b-5p	MIRT609587	GPM6B	-2.47	9.20E-26
hsa-miR-335-3p	MIRT609698	GFRA1	-3.38	4.44E-17
hsa-miR-335-3p	MIRT610269	PCSK2	-4.53	9.59E-27
hsa-miR-335-3p	MIRT615437	FAXC	-2.09	9.72E-08
hsa-miR-93-5p	MIRT615447	FAXC	-2.09	9.72E-08
hsa-miR-335-3p	MIRT616818	FGF10	-2.03	4.82E-07
hsa-miR-335-3p	MIRT618054	PCDH19	-2.15	1.17E-12
hsa-miR-335-3p	MIRT623191	MYOCD	-2.69	1.16E-13
hsa-miR-196a-3p	MIRT627264	XKR4	-3.14	6.35E-18
hsa-miR-93-5p	MIRT628565	MELK	2.59	2.19E-18
hsa-miR-181b-5p	MIRT633492	WDR72	3.49	1.46E-05
hsa-miR-181a-5p	MIRT633493	WDR72	3.49	1.46E-05
hsa-miR-18a-3p	MIRT638250	SLC16A9	-2.24	4.05E-09
hsa-miR-335-3p	MIRT638317	RNF150	-2.17	1.88E-10
hsa-miR-18a-3p	MIRT638737	FAXC	-2.09	9.72E-08
hsa-miR-335-3p	MIRT639081	ADCYAP1	-2.03	2.37E-06
hsa-miR-181b-3p	MIRT642414	CILP2	2.57	2.55E-07
hsa-miR-335-3p	MIRT642579	PCLAF	2.23	3.45E-15
hsa-miR-335-3p	MIRT650016	ADAMTS8	-2.04	1.81E-07
hsa-miR-93-5p	MIRT685721	BHMT2	-2.30	1.31E-14
hsa-miR-93-5p	MIRT694951	ANKS4B	2.11	3.66E-05
hsa-miR-93-5p	MIRT699286	SLC6A4	-2.76	2.22E-17
hsa-miR-146b-3p	MIRT704958	CBX2	2.17	2.10E-08
hsa-miR-196a-3p	MIRT708479	OLR1	2.89	5.14E-12
hsa-miR-335-3p	MIRT708630	STMN4	-2.02	2.87E-08
hsa-miR-18a-3p	MIRT709746	UBD	2.83	8.46E-08
hsa-miR-93-3p	MIRT709918	GRIK3	-3.64	2.69E-20
hsa-miR-146b-3p	MIRT710266	FAM107A	-2.91	9.59E-27
hsa-miR-18a-3p	MIRT710270	FAM107A	-2.91	9.59E-27
hsa-miR-196a-3p	MIRT711377	SKA1	2.16	1.59E-14
hsa-miR-18a-3p	MIRT711961	CYP27B1	2.17	1.17E-12
hsa-miR-93-3p	MIRT716717	SCN7A	-3.84	3.41E-19

© 2021 Izumi D et al. *JAMA Network Open.*

hsa-miR-93-3p	MIRT717236	SH2D5	2.04	7.45E-06
hsa-miR-146b-3p	MIRT717691	PTGS1	-2.10	8.56E-22
hsa-miR-146b-5p	MIRT717743	MYLK	-2.18	4.63E-11
hsa-miR-335-3p	MIRT718902	GALR1	-2.56	9.03E-12
hsa-miR-335-3p	MIRT722849	NEGR1	-2.56	6.06E-17
hsa-miR-93-5p	MIRT726903	POLQ	2.55	4.16E-17
hsa-miR-93-5p	MIRT726927	PKMYT1	2.62	1.86E-16
hsa-miR-181b-5p	MIRT727098	NCAPG	2.38	3.79E-16
hsa-miR-181a-5p	MIRT727100	NCAPG	2.38	3.79E-16
hsa-miR-181b-5p	MIRT732307	SPP1	3.09	1.75E-07
hsa-miR-18a-5p	MIRT732587	MYLK	-2.18	4.63E-11
hsa-miR-181b-5p	MIRT732607	TNFRSF11B	2.07	1.16E-05
hsa-miR-181b-5p	MIRT732608	TNFSF11	2.76	5.71E-14
hsa-miR-135b-5p	MIRT732742	ADAM12	3.21	4.85E-13
hsa-miR-196a-5p	MIRT732869	HOXA10	4.24	2.35E-09
hsa-miR-146b-5p	MIRT732960	NOVA1	-2.36	1.17E-12
hsa-miR-196a-5p	MIRT733362	MAMDC2	-4.28	8.61E-32
hsa-miR-21-3p	MIRT733453	HPGD	-2.95	1.79E-19
hsa-miR-135b-3p	MIRT733901	CCL7	2.70	1.84E-11
hsa-miR-21-5p	MIRT734186	MSLN	2.87	1.52E-04
hsa-miR-181a-5p	MIRT734362	PRKN	-2.13	1.60E-20
hsa-miR-196b-5p	MIRT734448	IGF2BP1	3.25	1.36E-04
hsa-miR-93-3p	MIRT734847	MMP3	3.45	1.53E-05
hsa-miR-135b-5p	MIRT734929	THBS2	2.10	1.68E-06
hsa-miR-146b-3p	MIRT734967	NPAS4	-2.53	1.10E-12
hsa-miR-146b-5p	MIRT735151	SLC5A5	-3.32	3.58E-10
hsa-miR-93-5p	MIRT735341	MMP3	3.45	1.53E-05
hsa-miR-181b-3p	MIRT738031	RBM20	-2.31	1.25E-12
hsa-miR-18a-3p	MIRT738296	SH2D5	2.04	7.45E-06
hsa-miR-335-3p	MIRT741342	HMGA2	2.93	3.84E-07
hsa-miR-93-3p	MIRT760677	CXCL5	2.39	9.01E-03
hsa-miR-18a-3p	MIRT763227	EPHA6	-2.86	2.30E-11
hsa-miR-196a-3p	MIRT763520	WT1	2.19	2.71E-04
hsa-miR-335-3p	MIRT765641	MAGEA12	3.37	3.86E-07
hsa-miR-21-3p	MIRT790183	RGS2	-2.06	2.04E-19

© 2021 Izumi D et al. *JAMA Network Open.*

eTable 3. Functional analysis of miRNA target genes identified 61 significantly enriched functional gene sets (BH-adjusted p-value < 0.0001)

Gene Set	Gene Ratio	*P* value	BH-adjusted *P* value
VECCHI GASTRIC CANCER EARLY DN	39/317	3.07E-22	1.99E-18
VECCHI GASTRIC CANCER EARLY UP	39/317	4.81E-20	1.56E-16
BOQUEST STEM CELL CULTURED VS FRESH UP	34/317	5.43E-16	8.81E-13
SABATES COLORECTAL ADENOMA DN	29/317	4.15E-16	8.81E-13
NABA SECRETED FACTORS	25/317	3.21E-11	1.90E-08
SMID BREAST CANCER NORMAL LIKE UP	24/317	1.40E-07	2.21E-05
POOLA INVASIVE BREAST CANCER UP	23/317	4.30E-11	2.32E-08
MIKKELSEN NPC HCP WITH H3K27ME3	23/317	9.24E-10	3.16E-07
RIGGI EWING SARCOMA PROGENITOR UP	23/317	8.44E-08	1.52E-05
GO SIGNAL RELEASE	23/317	2.38E-07	3.36E-05
HORIUCHI WTAP TARGETS DN	22/317	1.20E-09	3.89E-07
DUTERTRE ESTRADIOL RESPONSE 24HR UP	22/317	1.81E-09	5.09E-07
GO COLLAGEN CONTAINING EXTRACELLULAR MATRIX	22/317	1.11E-07	1.84E-05
GO EXTRACELLULAR STRUCTURE ORGANIZATION	22/317	2.07E-	3.05E-05

© 2021 Izumi D et al. *JAMA Network Open.*

		07	
ZHENG GLIOBLASTOMA PLASTICITY UP	21/317	4.71E-11	2.35E-08
GO G PROTEIN COUPLED RECEPTOR BINDING	21/317	5.09E-10	1.94E-07
FLORIO NEOCORTEX BASAL RADIAL GLIA DN	20/317	4.35E-12	3.53E-09
DELYS THYROID CANCER DN	20/317	1.80E-10	7.79E-08
AFFAR YY1 TARGETS DN	20/317	7.74E-10	2.79E-07
MEISSNER BRAIN HCP WITH H3K27ME3	20/317	1.52E-09	4.49E-07
GO COGNITION	20/317	9.69E-09	2.10E-06
ROSTY CERVICAL CANCER PROLIFERATION CLUSTER	19/317	1.51E-13	1.96E-10
KOBAYASHI EGFR SIGNALING 24HR DN	19/317	4.54E-09	1.13E-06
DURAND STROMA S UP	19/317	5.47E-08	1.07E-05
SENGUPTA NASOPHARYNGEAL CARCINOMA UP	19/317	7.93E-08	1.47E-05
GO HORMONE TRANSPORT	19/317	1.96E-07	2.96E-05
YANG BCL3 TARGETS UP	19/317	2.49E-07	3.36E-05
CHIANG LIVER CANCER SUBCLASS PROLIFERATION UP	18/317	1.12E-10	5.21E-08
GO CYTOKINE ACTIVITY	18/317	2.99E-09	8.04E-07

© 2021 Izumi D et al. *JAMA Network Open.*

GO CYTOKINE RECEPTOR BINDING	18/317	1.56E-07	2.41E-05
LEE EARLY T LYMPHOCYTE UP	17/317	2.87E-13	3.10E-10
GRAHAM CML DIVIDING VS NORMAL QUIESCENT UP	17/317	1.39E-09	4.30E-07
GO REGULATION OF HORMONE SECRETION	17/317	2.76E-07	3.58E-05
NABA CORE MATRISOME	17/317	5.18E-07	6.34E-05
VECCHI GASTRIC CANCER ADVANCED VS EARLY UP	16/317	4.98E-09	1.16E-06
NAKAYAMA SOFT TISSUE TUMORS PCA2 UP	15/317	2.28E-12	2.11E-09
KONG E2F3 TARGETS	15/317	7.15E-12	5.15E-09
SOTIRIOU BREAST CANCER GRADE 1 VS 3 UP	15/317	6.37E-09	1.43E-06
GO MYELOID LEUKOCYTE MIGRATION	15/317	2.55E-07	3.37E-05
GO LEUKOCYTE CHEMOTAXIS	15/317	6.74E-07	7.61E-05
GO GLYCOSAMINOGLYCAN BINDING	15/317	8.01E-07	8.81E-05
ANDERSEN CHOLANGIOCARCINOMA CLASS2	14/317	2.44E-07	3.36E-05
GRAHAM NORMAL QUIESCENT VS NORMAL DIVIDING DN	13/317	4.38E-10	1.77E-07
LE EGR2 TARGETS UP	13/317	5.03E-09	1.16E-06

© 2021 Izumi D et al. *JAMA Network Open.*

WILCOX RESPONSE TO PROGESTERONE UP	13/317	3.29E-07	4.18E-05
VART KSHV INFECTION ANGIOGENIC MARKERS UP	13/317	6.35E-07	7.35E-05
WINNEPENNINCKX MELANOMA METASTASIS UP	13/317	6.35E-07	7.35E-05
GO HEPARIN BINDING	13/317	6.81E-07	7.61E-05
KANG DOXORUBICIN RESISTANCE UP	12/317	1.20E-11	7.78E-09
NAKAYAMA SOFT TISSUE TUMORS PCA2 DN	12/317	3.10E-09	8.04E-07
TURASHVILI BREAST LOBULAR CARCINOMA VS LOBULAR NORMAL UP	12/317	1.00E-08	2.10E-06
WHITEFORD PEDIATRIC CANCER MARKERS	12/317	1.09E-07	1.84E-05
ZHOU CELL CYCLE GENES IN IR RESPONSE 24HR	12/317	2.28E-07	3.29E-05
GAVIN FOXP3 TARGETS CLUSTER P6	11/317	7.41E-08	1.41E-05
CROONQUIST IL6 DEPRIVATION DN	11/317	1.16E-07	1.88E-05
GO CHEMOKINE RECEPTOR BINDING	10/317	2.79E-08	5.65E-06
RHODES UNDIFFERENTIATED CANCER	9/317	5.99E-07	7.20E-05
GO SOMATIC STEM CELL POPULATION MAINTENANCE	9/317	8.67E-07	9.21E-05
GO CHEMOKINE ACTIVITY	8/317	3.69E-07	4.61E-05
REICHERT MITOSIS LIN9 TARGETS	7/317	1.05E-	1.84E-05

© 2021 Izumi D et al. *JAMA Network Open.*

		07	
GO DOPAMINERGIC NEURON DIFFERENTIATION	7/317	8.19E-07	8.85E-05

© 2021 Izumi D et al. *JAMA Network Open.*

eTable 4. Comparison between the expression of the 10 miRNAs in GC and normal frozen tissues

miRNA	AUC*	aveTumor**	aveNormal**	log2FC***	*P* value
miR-21	0.72	5.58	4.62	0.96	7.3E-05
miR-196a	0.93	-2.99	-7.20	4.21	8.8E-14
miR-146b	0.59	1.19	0.61	0.58	5.4E-02
miR-196b	0.89	-5.71	-9.24	3.53	1.5E-11
miR-135b	0.40	-5.59	-4.84	-0.75	9.6E-01
miR-181b	0.60	-0.02	-0.43	0.41	4.5E-02
miR-181a	0.58	-1.61	-2.00	0.39	7.5E-02
miR-93	0.60	0.49	0.14	0.36	4.1E-02
miR-335	0.62	-4.05	-4.44	0.38	1.7E-02
miR-18a	0.77	-2.15	-3.36	1.22	1.3E-06
*AUC, area under the ROC curve. **ave, average. ***FC, fold change					

© 2021 Izumi D et al. *JAMA Network Open.*

eTable 5. Statistical evaluation of the diagnostic value of the miRNAs in differentiating GC patients from healthy participants in the serum internal validation cohort

	AUC	Odds Ratio	Specificity	Sensitivity	Accuracy
miR-18a	0.67 (0.57 - 0.76)	4.24 (2.09 - 8.57)	0.44 (0.30 - 0.86)	0.84 (0.49 - 0.95)	0.78 (0.54 - 0.86)
miR-93	0.67 (0.58 - 0.75)	3.66 (1.83 - 7.35)	0.67 (0.49 - 0.95)	0.64 (0.34 - 0.86)	0.64 (0.44 - 0.79)
miR-146b	0.58 (0.50 - 0.66)	2.65 (1.27 - 5.53)	0.74 (0.63 - 1.00)	0.48 (0.19 - 0.62)	0.52 (0.32 - 0.63)
miR-181b	0.80 (0.74 - 0.85)	15.62 (5.39 - 45.32)	0.91 (0.74 - 1.00)	0.62 (0.50 - 0.81)	0.66 (0.58 - 0.81)
miR-335	0.66 (0.59 - 0.74)	7.10 (2.45 - 20.57)	0.91 (0.51 - 0.98)	0.42 (0.36 - 0.81)	0.50 (0.45 - 0.78)
Signature	0.90 (0.85 - 0.94)	20.69 (8.62 - 49.67)	0.84 (0.77 - 1.00)	0.80 (0.60 - 0.90)	0.81 (0.66 - 0.88)
Abbreviation: AUC, area under the ROC curve. OR, odds ratio.					

© 2021 Izumi D et al. *JAMA Network Open.*

eTable 6. Univariate and multivariate analysis using clinical factors and the circulating miRNA signature

	**Univariate analysis**			**Multivariate analysis**		
MicroRNA	OR (95% CI)	*P* value		OR (95% CI)	*P* value	
Age	2.22 (1.09 - 4.50)	**0.01**		1.03 (1.00 - 1.06)	0.05	
Gender	1.32 (0.68 - 2.56)	0.58		0.51 (0.19 – 1.28)	0.17	
CEA	0.36 (0.14 - 0.95)	0.94		0.99 (0.97 - 1.00)	0.19	
CA19-9	0.51 (0.19 - 1.35)	0.77		1.00 (1.00 – 1.01)	0.02	
Signature	20.69 (8.62 - 49.67)	**<0.01**		2.96 (2.10 – 4.52)	**<0.01**	
Abbreviation: OR, odds ratio. CI, confidence interval						

© 2021 Izumi D et al. *JAMA Network Open.*

eTable 7. Analyses comparing our 5-circulating-miRNA signature with CEA and CA19-9 for non-invasive detection of gastric cancer (GC) across all stages and stage I in the prospective validation cohort.

Comparison	Cohort	No. of participants	AUC	Accuracy	Sensitivity<br>Specificity	PPV<br>NPV	Difference
**miRNA signature for various patients**							
Gastric cancer	Kumamoto	216 vs. 43	0.90 (0.85–0.94)	0.81 (0.76–0.86)	0.80 (0.60–0.90)<br>0.84 (0.77–1.00)	0.96 (0.76–0.85)<br>0.46 (0.39–0.54)	
Gastric cancer	Kumamoto and Nagoya	504 vs. 82	0.81 (0.76-0.85)	0.68 (0.64-0.72)	0.65 (0.61-0.69)<br>0.84 (0.76-0.91)	0.96 (0.94-0.98)<br>0.28 (0.25-0.31)	
Colorectal cancer	Kumamoto	38 vs. 43	0.54 (0.41–0.67)	0.60 (0.51–0.70)	0.39 (0.11–0.68)<br>0.79 (0.58–1.00)	0.62 (0.45–0.80)<br>0.60 (0.53–0.67)	***P*<0.05**
Colorectal cancer	GSE25609	20 vs. 21	0.58 (0.39-0.76)	0.63 (0.56-0.73)	0.25 (0.10-0.45)<br>1.00 (1.00-1.00)	1.00 (1.00-1.00)<br>0.58 (0.54-0.66)	***P*<0.05**
Pancreatic cancer	GSE85589	88 vs. 19	0.63 (0.48-0.77)	0.71 (0.62-0.79)	0.74 (0.64-0.82)<br>0.58 (0.37-0.79)	0.89 (0.84-0.94)<br>0.32 (0.21-0.44)	***P*<0.05**
Non-small cell lung cancer	GSE46729	24 vs. 24	0.48 (0.31-0.65)	0.56 (0.48-0.65)	0.96 (0.88-1.00)<br>0.17 (0.04-0.33)	0.53 (0.49-0.59)<br>0.83 (0.33-1.00)	***P*<0.05**
Breast cancer	GSE31309	48 vs. 57	0.61 (0.50-0.72)	0.59 (0.51-0.67)	0.90 (0.79-0.98)<br>0.33 (0.23-0.46)	0.53 (0.48-0.58)<br>0.79 (0.64-0.94)	***P*<0.05**
Ovarian cancer	GSE31568	15 vs. 70	0.56 (0.42-0.71)	0.40 (0.32-0.48)	1.00 (1.00-1.00)<br>0.27 (0.17-0.37)	0.23 (0.21-0.25)<br>1.00 (1.00-1.00)	***P*<0.05**
Stage I GC	Kumamoto	84 vs. 43	0.89 (0.83–0.94)	0.80 (0.72–0.87)	0.77 (0.58–0.94)<br>0.84 (0.65–1.00)	0.90 (0.84–0.96)<br>0.66 (0.57–0.76)	
**Comparing with current tumor markers**							
Signature	Kumamoto	216 vs. 43	0.90 (0.85–0.94)	0.81 (0.76–0.86)	0.80 (0.60–0.90)<br>0.84 (0.77–1.00)	0.96 (0.76–0.85)<br>0.46 (0.39–0.54)	
CEA (cutoff 3.4 ng/mL)	Kumamoto	151 vs. 24	0.55 (0.40–0.70)	0.77 (0.70–0.83)	0.83 (0.34–0.97)<br>0.36 (0.14–0.86)	0.90 (0.87–0.93)<br>0.24 (0.12–0.38)	***P*<0.01**
CA19-9 (cutoff 37.0 U/mL)	Kumamoto	148 vs. 21	0.56 (0.42–0.70)	0.59 (0.51–0.66)	0.59 (0.14–0.94)<br>0.58 (0.21–1.00)	0.91 (0.87–0.96)<br>0.15 (0.10–0.21)	***P*<0.01**

Range values surrounded by brackets mean 95% confidence intervals. Abbreviations: AUC, area under the ROC curve. PPV, positive predictive value. NPV, negative predictive value. CEA, carcinoembryonic antigen. CA19-9, cancer antigen 19-9.

© 2021 Izumi D et al. *JAMA Network Open.*

eTable 8. Comparison of the performance of our 3-circulating-miRNA signature against CEA and CA19-9 for non-invasive detection of all-stage GC and stage I GC in the prospective validation cohort.

	**3-miRNA signature** 216 (GC) vs 43 (Healty)	**3-miRNA signature (stage I)** 84 (GC) vs 43 (Healty)	**CEA (all stage)** 151 (GC) vs 24 (Healty)	**CA19-9 (all stage)** 148 (GC) vs 21 (Healty)
AUC	0.86 (0.83–0.90)	0.85 (0.79–0.91)	0.65 (0.57–0.73)	0.67 (0.59–0.76)
Odds Ratio	18.2 (10.4–32.0)	18.8 (8.89–39.9)	2.83 (1.57–5.12)	0.14 (0.07–0.27)
Accuracy	0.80 (0.75–0.84)	0.84 (0.79–0.89)	0.63 (0.57–0.70)	0.73 (0.67–0.79)
Sensitivity	0.72 (0.65–0.78)	0.72 (0.59–0.83)	0.57 (0.46–0.68)	0.56 (0.45–0.68)
Specificity	0.88 (0.83–0.92)	0.88 (0.83–0.92)	0.68 (0.59–0.77)	0.85 (0.78–0.91)
PPV	0.86 (0.80–0.91)	0.65 (0.56–0.75)	0.57 (0.49–0.65)	0.73 (0.63–0.83)
NPV	0.75 (0.71–0.80)	0.91 (0.88–0.94)	0.69 (0.62–0.75)	0.74 (0.69–0.79)

Abbreviations: GC, gastric cancer. Healthy, healthy participants. CEA, carcinoembryonic antigen. CA19-9, cancer antigen 19-9. AUC, area under the ROC curve. PPV, positive predictive value. NPV, negative predictive value.

© 2021 Izumi D et al. *JAMA Network Open.*

eTable 9. Univariate and multivariate analyses comparing our 3-circulating-miRNA signature with age, sex, CEA, and CA19-9 for non-invasive detection of GC across all stages and stage I in the prospective validation cohort.

	**Prospective Validation Cohort (All Stages)**					**Prospective Validation Cohort (Stage I)**			
	**Univariable**		**Multivariable**			**Univariable**		**Multivariable**	
	OR (95% CI)	*P*	OR (95% CI)	*P*		OR (95% CI)	*P*	OR (95% CI)	*P*
3-miR	18.2 (10.4–32.0)	1.83E-35	8.59 (4.56–18.2)	8.36E-10		18.8 (8.89–39.9)	4.16E-18	4.25 (2.08–10.1)	2.95E-4
Age	0.20 (0.12–0.33)	8.88E-2	1.00 (0.97–1.03)	8.47E-1		0.17 (0.09–0.33)	2.36E-1	1.03 (0.99–1.07)	1.58E-1
Sex	1.33 (0.85–2.09)	2.10E-1	0.82 (0.35–1.84)	6.31E-1		1.95 (0.96–3.99)	5.62E-2	1.85 (0.50–7.98)	3.74E-1
CEA	2.83 (1.57–5.12)	5.90E-6	1.42 (1.09–1.98)	2.64E-2		3.37 (1.35–8.38)	1.20E-2	2.01 (1.27–3.36)	4.41E-3
CA19-9	0.14 (0.07–0.27)	3.37E-1	1.00 (0.99–1.02)	2.24E-1		0.07 (0.03–0.18)	1.44E-4	0.86 (0.77–0.94)	2.52E-3

Abbreviations: OR, odds ratio, CEA, carcinoembryonic antigen. CA19-9, cancer antigen 19-9.
Healthy participants 173, patients with all stages GC 176 and patients with stage I GC 54.

© 2021 Izumi D et al. *JAMA Network Open.*

eTable 10. Results of cost-effectiveness analysis

	**Non-invasive screening program**	**No-screening**
Cohort size	100000	100000
Total number of cancer patients in the cohort	5000	5000
Compliance	45%	10%
Stage of diagnosis (Stage 1; 2; 3; 4)	17.57%; 21.96%; 34.74%;25.73%	11.50%; 19.47%; 42.71%; 26.33%
Average cost of diagnosis	CNY 447.1	CNY 48.7
**Results of cost-effectiveness analysis (CEA)**		
Total cost	1172.2M	1115.1M
Total QALY	2337.0K	2333.5K
Cost difference	570.1	
Effect difference	0.035	
Cost of saving 1 QALY	CNY 16162.5/QALY	

© 2021 Izumi D et al. *JAMA Network Open.*

eTable 11. Clinical assumptions of the hypothetical cohort used for the cost effectiveness analysis

	**Value**	**Ref.**
**Cost**		
miRNA assay	300	Estimated
Endoscopy	304	Estimated using internal records
Biopsy	108	Estimated using internal records
Stage 1 treatment	62205	Annual cost Yang et al. Chinese Journal of Cancer Research 2018 (PMID: 30210224)
Stage 2 treatment	42231	
Stage 3 treatment	49983	
Stage 4 treatment	36865	
Follow-up examinations	700	Estimated
Staging Investigation	1500	Estimated
**Cancer cases in high risk population**	5%	Estimated
**Portion by stage**		
Stage 1	11.50%	Yang et al. Chinese Journal of Cancer Research 2018 (PMID: 30210224)
Stage 2	19.47%	
Stage 3	42.71%	
Stage 4	26.33%	
**Incidence of cancer**	(1/100000)	
50-54	29.31	Yang et al. Chinese Journal of Cancer Research 2018 (PMID: 30046223)
55-59	54.36	
60-64	87.09	
65-69	122.39	
70-74	156.03	
>75	513.72	
**5-yr recurrence rate of cancer by stage**		
Stage 1	2.19%	Xu et al. Cancer Medicine 2016 (PMID: 32420703)
Stage 2	8.12%	
Stage 3	28.35%	
**Utility Values**		
Stage 1	0.88	Zhou el at. PLoS ONE 2013 (PMID: 24386314)
Stage 2	0.86	
Stage 3	0.77	
Stage 4	0.68	
Cured	0.88	Estimated

© 2021 Izumi D et al. *JAMA Network Open.*
